# Modeling galvanostatic charge–discharge of nanoporous supercapacitors

**DOI:** 10.1038/s43588-021-00153-5

**Published:** 2021-11-22

**Authors:** Liang Zeng, Taizheng Wu, Ting Ye, Tangming Mo, Rui Qiao, Guang Feng

**Affiliations:** 1 State Key Laboratory of Coal Combustion, School of Energy and Power Engineering, Huazhong University of Science and Technology (HUST), Wuhan, China; 2 Department of Mechanical Engineering, Virginia Tech, Blacksburg, VA USA

**Keywords:** Supercapacitors, Energy, Atomistic models, Molecular dynamics

## Abstract

Molecular modeling has been considered indispensable in studying the energy storage of supercapacitors at the atomistic level. The constant potential method (CPM) allows the electric potential to be kept uniform in the electrode, which is essential for a realistic description of the charge repartition and dynamics process in supercapacitors. However, previous CPM studies have been limited to the potentiostatic mode. Although widely adopted in experiments, the galvanostatic mode has rarely been investigated in CPM simulations because of a lack of effective methods. Here we develop a modeling approach to simulating the galvanostatic charge–discharge process of supercapacitors under constant potential. We show that, for nanoporous electrodes, this modeling approach can capture experimentally consistent dynamics in supercapacitors. It can also delineate, at the molecular scale, the hysteresis in ion adsorption–desorption dynamics during charging and discharging. This approach thus enables the further accurate modeling of the physics and electrochemistry in supercapacitor dynamics.

## Abstract (web summary)

The authors propose a molecular modeling approach to simulating the galvanostatic charge–discharge process of supercapacitors under constant-potential conditions. This model can accurately predict supercapacitor dynamics when compared with experimental observations.

## Main

Supercapacitors have been attracting much attention because of their high power densities and superior cycle times^1,2^. Researchers often resort to molecular modeling to investigate the thermodynamic and kinetic behavior of supercapacitors, particularly those with nanoporous electrodes, because molecular simulations can provide a precise microscopic picture of electric double layers (EDLs) and their formation in supercapacitors^3–5^. In the molecular modeling of supercapacitors, how to describe electrode polarization is a critical issue. The most straightforward way is to distribute charges uniformly on the electrode atoms in the constant charge method (CCM)^4–6^, whereas the constant potential method (CPM), an approach that is computationally expensive but can more precisely model realistic conditions, maintains electrode atoms at constant potentials by self-consistently adjusting the electrode charges based on the electrode potential and ionic environment^7–12^. To explore the equilibrium performance of supercapacitors, CCM can be used to approximate systems with open electrodes (where the electrode surface is in contact with the bulk electrolyte^13^, for example, a planar^14^, cylindrical^6^ or spherical^15^ surface), but it is not accurate for nanoporous electrodes^10–12,16,17^. To model the charge–discharge process of nanoporous supercapacitors, CPM is preferred because its self-consistent adjustment of electrode charges, absent in CCM, can produce the correct charging dynamics and heat generation^17,18^.

When studying the charging and discharging processes of supercapacitors, early CPM simulations often adopted the potential control mode, in which step-like^7–12^, linear^19^ or climbing-type^20^ potential differences are applied between the positive and negative electrodes. Molecular simulations in the potential control mode help to understand the fundamentals of charging and discharging supercapacitors. However, they cannot offer insights into the charging kinetics in the galvanostatic mode (that is, when the electrode is applied with a constant electric current^21^), which has been widely used in practical applications^22^ and fundamental electrochemical studies (for example, galvanostatic charge–discharge, GCD)^23,24^. To simulate the charging and discharging process of supercapacitors that are under more realistic conditions (for example, the current control with porous electrodes), it is desirable to have advanced modeling approaches that are able to regulate the electric current (or galvanostatic) and maintain a constant potential across each atom within the electrode. Most recently, an approach combining finite electric displacement and a constant potential technique was developed for this purpose^25^. This approach has been applied to open-electrode systems, but has yet to be demonstrated for nanoporous systems. As such, existing GCD simulations have largely been based on CCM, with the equivalent charge set on each electrode atom and varied linearly with time (in the following, this method is termed GCD-CCM)^26,27^. However, due to the lack of self-consistent adjustment of electrode charges, the accuracy of GCD-CCM, especially for nanoporous systems, remains to be delineated.

**Fig. 1:**
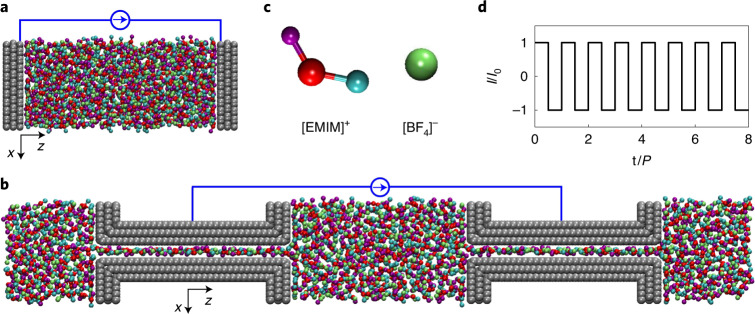
Molecular simulations of the GCD of supercapacitors. **a**,**b**, Snapshots of supercapacitors featuring open electrodes (**a**) and nanoporous electrodes (**b**). **c**, Coarse-grained models of the ionic liquid [EMIM][BF_4_] used in the supercapacitors. **d**, Applied electric current with time (*t*) during GCD.

Based on previous CPM studies^7,8,12^, we developed a method to model the GCD of supercapacitors under constant potential, rigorously enforcing the equipotential state within an electrode at each time step (named GCD-CPM; Methods). Both GCD-CPM and GCD-CCM were applied to molecular dynamics (MD) simulations of two typical supercapacitor systems: open electrode (Fig. 1a) and nanoporous electrode (Fig. 1b). The ionic liquid ethyl-3-methyl-imidazolium tetrafluoroborate ([EMIM][BF_4_]) was used as the electrolyte in both systems (Fig. 1c). After equilibrating the simulation under the non-charged state (potential of zero surface charge), we applied a square-wave current with amplitude *I*_0_ and period *P* (Fig. 1d and Methods). We then analyzed the time evolution of the electrode potential and EDL formation in both systems. We further demonstrated the effectiveness of GCD-CPM via experimental validation.

## Results

### GCD-CPM and GCD-CCM for open-electrode systems

**Fig. 2:**
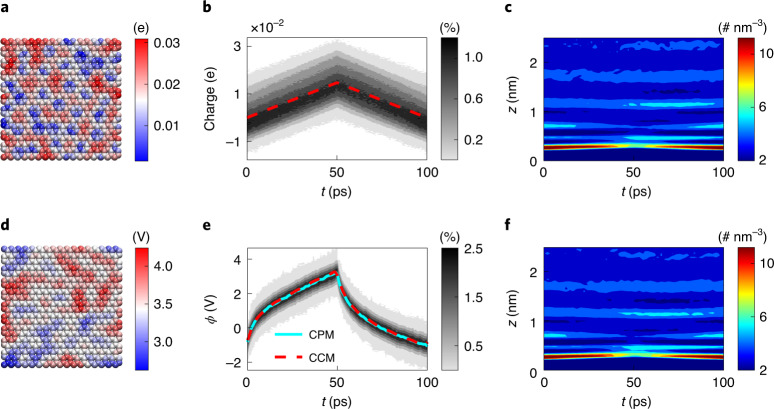
GCD of open-electrode systems. **a**–**c**, GCD-CPM modeling: a snapshot of the positive electrode at 50 ps in the MD simulation, with atoms colored by their instantaneous charges (**a**); the probability distributions of electrode charges varying with time (gray contour) and their average (dashed line) (**b**); evolution of ion number density with time (*t*) and distance (*z*) from the electrode (**c**). **d**–**f**, GCD-CCM modeling: a snapshot of the positive electrode at 50 ps, with atoms colored by their instantaneous potential (**d**); the probability distributions of the electrode potentials (gray contour) and their average (red dashed line) (**e**); evolution of ion number density (**f**). For comparison, the solid cyan line in **e** is the electrode potential obtained by GCD-CPM. The error bar for the electrode potential is close to the line width and is thus not shown. Source data

We first compared GCD-CPM and CGD-CCM in open-electrode systems. Like the GCD test in the experiments^23,24^, MD simulation systems were charged and discharged with a square-wave current, becoming stable after a few cycles (details are provided in Supplementary Section 1). Figure 2 shows the modeling-obtained GCD of a stable cycle with a period of 100 ps. In molecular modeling with GCD-CPM, the charges distributed on the electrode atoms are not uniform at any instant (Fig. 2a) due to the thermal motion of the electrolyte molecules^5,28^, as in previous works using CPM with step-like potentials^4,5,28^. Such a non-uniform distribution can be quantified by the probability distribution of the charges on the electrode atoms^5,28^. These charge probability distributions were analyzed in GCD-CPM simulations for each time step during the charge–discharge process (see Methods for the detailed probability distribution calculation). They have a volcano-like shape, and their width (or variance) changes little with time (Fig. 2b shows results for the positive electrode and Supplementary Fig. 2a for the negative electrode) in response to the applied current (Fig. 1d). On the electrolyte side, cations and anions, driven by the electrode polarization, form dynamic EDLs, which are delineated by the ion number density as a function of time and distance from the electrode surface (Fig. 2c and Supplementary Figs. 3 and 4). It is found that EDLs have typical layering structures, consistent with previous findings^4,29,30^. During the charging process (0–50 ps), the first cation layer decreases with time and moves slightly away from the electrode surface, and this trend is reversed during discharging (50–100 ps).

**Extended Data Fig. 1:**
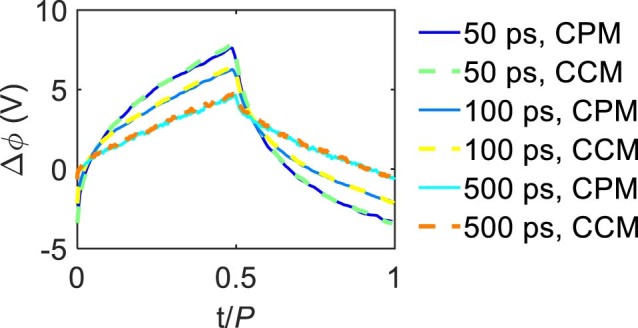
GCD curves with various periods for open electrode systems. GCD curves are obtained from GCD-CPM and GCD-CCM simulations. Source data

The GCD of an open-electrode system was also explored using GCD-CCM. Instead of adjusting the charges distributed on electrode atoms at each time step to keep the electrode in an equipotential state in GCD-CPM modeling, in GCD-CCM modeling, the charge on each electrode atom is preset with the same value. The electrode potential is computed accordingly, showing a distinctly non-uniform distribution (Fig. 2d). However, the averaged potential over electrode atoms at each time closely matches the results obtained from the GCD-CPM calculation (Fig. 2e and Supplementary Fig. 2b). The evolution of cation density at the positive electrode obtained by GCD-CCM calculations was also found to be nearly the same as that obtained by GCD-CPM (Fig. 2c,f and Supplementary Fig. 3), and a comparison of more dynamic EDL structures reaches the same agreement (Supplementary Fig. 4). Besides the number density, ion orientation is also an important descriptor to characterize the EDL. On further examining the ion orientation, the angular distributions of the cations adsorbed on the negative electrode, obtained by these two methods, were almost the same (Supplementary Fig. 5). With additional electric currents with different amplitudes and periods, the results obtained using these two methods are also consistent (Extended Data Fig. 1).

### Importance of GCD-CPM for nanoporous-electrode systems

**Fig. 3:**
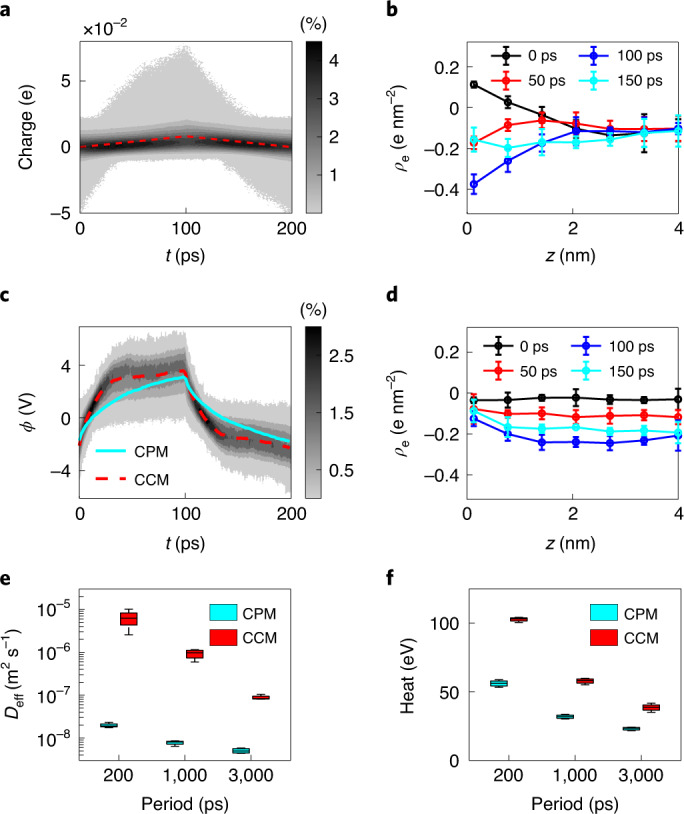
GCD-CPM and GCD-CCM modeling of GCD in nanoporous-electrode systems. **a**, The gray contour shows the probability distributions of atom charges on the positive electrode, obtained from GCD-CPM simulations, and the dashed line is the average of such charges. **b**, Evolution of in-pore charge density from the electrolyte along the pore (where *z* = 0 is the pore entrance), obtained from GCD-CPM simulations. **c**, The gray contour indicates the probability distributions of potentials on the positive electrode, obtained from GCD-CCM simulations. The red dashed line is the average of the potentials and the solid green line is the potential obtained from GCD-CPM simulations. **d**, Evolution of the in-pore charge density from the electrolyte, obtained from GCD-CCM simulations. **e**, Effective diffusivity of the in-pore electrolyte under positive polarization. **f**, Heat generation of a GCD cycle. The pore width is 0.67 nm. Error bars in **b** and **d** indicate 1 s.d. of four independent simulations. The box plots in **e** and **f** represent the maximal, minimal, median, 25% and 75% of the data distribution. Source data

**Extended Data Fig. 2:**
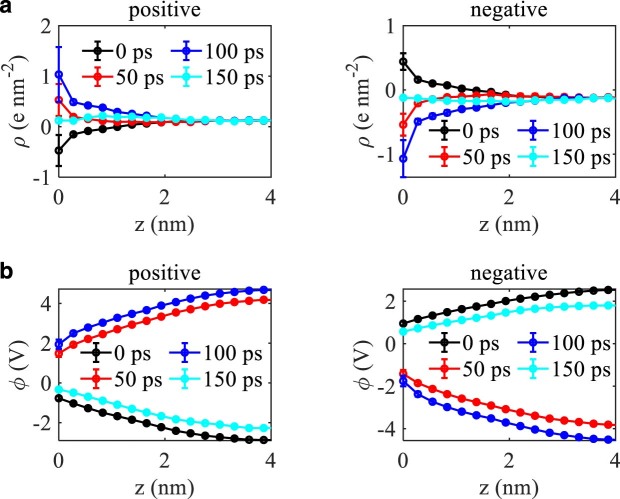
Distributions of charge and potential on electrodes atoms in nanoporous electrode systems. **a**, Spatial distributions of electrode atom charges along pore axis for GCD-CPM simulations. **b**, Spatial distributions of electrode atom potentials along pore axis for GCD-CCM simulations. The data of each time counts the data of 5 ps around it. Error bars indicate one standard deviation of 4 independent simulations. Source data

We next investigated the GCD of nanoporous-electrode systems. In GCD-CPM modeling, the probability distributions of electrode charges are more non-uniform and can vary more significantly with time (Fig. 3a and Supplementary Fig. 6a), compared with those in open-electrode systems (Fig. 2b and Supplementary Fig. 2a). This can be ascribed to the non-uniform distribution of electrode charges along the pore axis (Extended Data Fig. 2a). In particular, the variation of surface charges at the pore entrance is more extensive than in the central portion of the pore. Driven by electrode polarization, the in-pore charge density *ρ*_e_, stemming from the electrolyte, exhibits a trend similar to the variation in electrode charges; that is, the change in charge density at the pore entrance is greater than at the center of the pore (Fig. 3b versus Extended Data Fig. 2a).

The potential distributions on the nanoporous electrodes were examined using GCD-CCM (Fig. 3c and Supplementary Fig. 6b) and compared to GCD-CPM. The potential was found to be more non-uniform than that distributed on the open electrode, and its average differs from the GCD-CPM results. Remarkably, there is a parabolic-like distribution of the electrode potential from the entrance to the central portion of a pore (Extended Data Fig. 2b), which conflicts with the fact that the electrode should be equipotential in reality, suggesting that GCD-CCM could result in an unphysical phenomenon. For GCD-CCM, the in-pore charges, driven by the uniformly distributed electrode charges, vary less than those obtained using GCD-CPM (Fig. 3d versus Fig. 3b).

Ion transport under nanoconfinement has been found to have a strong correlation with the charging dynamics and power density of nanoporous supercapacitors^1,3,11^. The in-pore ion transport during the charge–discharge process can be evaluated by means of the effective diffusivity, *D*_eff_, which is described as^11,31^

1 $$\frac{{\partial \rho _{\rm{e}}}}{{\partial t}} = D_{\rm{eff}}\frac{{\partial \rho _{\rm{e}}^2}}{{\partial ^2z}},$$

where *ρ*_e_ is the charge density of the in-pore electrolyte, *t* is time and *z* is the location along the pore axis. Using equation (1) and the evolution of the in-pore charge density (Fig. 3 and Supplementary Fig. 7), the effective diffusivity of the electrolyte in positive nanoporous electrodes was calculated and is shown in Fig. 3e (calculation details are provided in the  Methods section). Using GCD-CPM modeling, for different periods *P*, the effective diffusivity in the pore of the positive electrode relative to the ion self-diffusion in the bulk electrolyte, *D*_eff_/*D*_bulk_, is 52 (*P* *=* 200 ps), 21 (*P* *=* 1,000 ps) and 13 (*P* *=* 3,000 ps), close to the results predicted by theory^11,31^ and molecular simulations with step-like CPM^11^. For GCD-CCM modeling, *D*_eff_/*D*_bulk_ is 16,566 (*P* = 200 ps), 2,434 (*P* *=* 1,000 ps) and 231 (*P* *=* 3,000 ps), considerably larger than the results from GCD-CPM. A similar trend is found for negative nanoporous electrodes (Supplementary Figs. 8 and 9).

Previous work reported that CCM modeling gives a large value for heat generation during the charging process, contradicting the Joule effect^17^. We investigated the heat generated during the charge–discharge process in nanoporous supercapacitors, calculating it as the energy removed from the system by a thermal bath (details of the heat calculation are provided in the Methods section). Figure 3f and Supplementary Fig. 10 show the total heat generation of a charge–discharge cycle and heat generation varying with time, respectively, demonstrating that the value of heat generation computed by GCD-CCM is much larger than that obtained from GCD-CPM calculations. Accordingly, GCD-CCM cannot be used for modeling the dynamics of nanoporous supercapacitors, at least for the nanoporous electrodes studied here.

### Experimental validation of GCD-CPM modeling

**Fig. 4:**
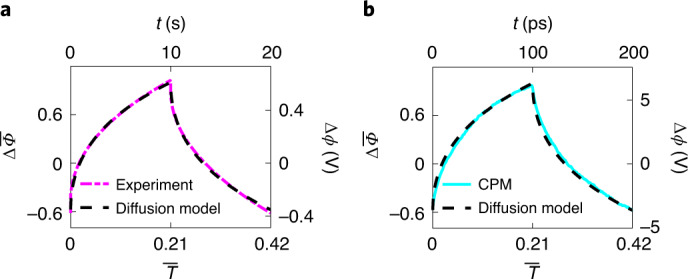
Comparison of GCD curves obtained from experiment and modeling. **a**, GCD curve in the experiment with a period of 20 s and its fitting by the dimensionless diffusion model. **b**, GCD curve of GCD-CPM with a period of 200 ps and its fitting by the dimensionless diffusion model. Source data

We next validated the molecular modeling with GCD-CPM through experimental galvanostatic measurements (experimental details are provided in the Methods section). The supercapacitor uses the same electrolyte as modeled in our simulation. The nanoporous carbon electrode has an average pore size very close to that in the molecular models. In the experiment, the GCD curve includes an ohmic drop caused by the electrode resistance (Supplementary Fig. 12)^23,32^. Because the electrode resistance is neglected in molecular modeling, the ohmic drop was removed from the experimental GCD curves, as described in the Methods section. This treatment aids a comparison of the experiment and modeling, but does not change the charge–discharge process of the nanoporous supercapacitors (Fig. 4a and Supplementary Fig. 12a).

The charging dynamics of the nanoporous electrodes can be explained by the diffusion model, which is rooted in mean-field theory^11,31^. To compare macroscale experiments and nanoscale modeling, we derive the dimensionless form of the diffusion model for the charging and discharging process of the nanoporous supercapacitors. The dimensionless diffusion model shows that the GCD curve, described by the dimensionless potential ${{{\mathrm{{\Delta}}}\bar {\varPhi}}}$ varying with dimensionless time ${{{\bar{T}}}}$, is only determined by the dimensionless period *α* (defined as the ratio of the electric current period to the system charging time constant). Details of the dimensionless diffusion model are provided in the Methods section. As shown in Fig. 4a, the diffusion model provides a good fit for the experimental GCD data, with a dimensionless period of 0.42. It was then used to fit the GCD curve, also with period *α* = 0.42, obtained by GCD-CPM modeling of nanoporous systems (Fig. 4b), and this also shows good agreement. Because the GCD curves in the experiment and modeling have the same dimensionless period, their fitted curves are the same, with actual periods in the experiment and modeling of 20 s and 200 ps, respectively. Therefore, with regulation of the electric current period, the GCD-CPM modeling is consistent with the experiment (Supplementary Fig. 13). However, the fitting reveals an apparent discrepancy between the experiment and GCD-CCM modeling (Supplementary Fig. 14). Accordingly, only GCD-CPM could predict the correct dynamic process for the charge–discharge of the nanoporous electrodes. Furthermore, the capacitances extracted from the GCD curves obtained from molecular simulations with GCD-CPM are compatible with those from our experimental measurements (details are provided in the Methods section and Supplementary Fig. 15), and both are consistent with previous experiments with the similar porous carbon electrodes and the same electrolyte^33,34^.

### Hysteresis in the charge–discharge process

**Fig. 5:**
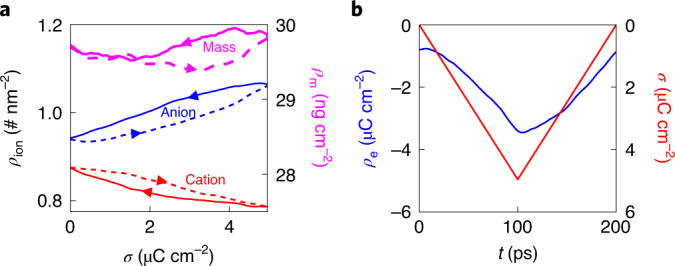
Hysteresis in nanoporous-electrode systems. **a**, The mass density of the in-pore electrolyte (magenta line) and the number densities of the in-pore cations and anions (red and blue lines, respectively). The solid lines show the discharging process and the dashed lines the charging process. **b**, The charge density coming from the in-pore electrolyte (blue line) and the charge density on the electrode (red line). The shown results are for the positive electrode. Source data

Figure 5a shows the hysteresis of the ion adsorption–desorption during the charge–discharge process of nanoporous supercapacitors, demonstrated by the time evolution of the in-pore electrolyte density. Specifically, in the positive porous electrode, driven by the applied current, counterions (that is, anions) are attracted into pores during charging and rejected out of the pores during discharging, while a reverse trend exists for co-ions (that is, cations). The desorption of the counterions during discharging does not backtrack with the adsorption during charging (that is, at the same surface charge, *σ*, the number of counterions during charging is different from that during discharging), and the change of co-ions in the desorption process is also different from that in the adsorption process. These findings indicate that there is a hysteresis in the ion response during the charge–discharge process, which is similar to the hysteresis loop experimentally observed in porous electrode supercapacitors probed by an electrochemical quartz crystal microbalance (note that the areal densities shown in our work are based on the specific surface area of the porous electrode rather than the area of quartz-crystal surface used in experiments)^35,36^.

The hysteresis of the ion adsorption–desorption can be understood by comparing the charges from the in-pore electrolyte and on the electrode (Fig. 5b). The change in the charge from the in-pore electrolyte exhibits a trend (increase or decrease with time) that is essentially the same as that on the electrode, but the rate of change on the electrolyte side is slower than that on the electrode side. This indicates that the ion transport in the electrolyte, especially under nanoconfinement, is sluggish compared to the charge transport through the electrode. As shown in Fig. 5b, at the beginning of a stable cycle at the positive electrode (at 0 ps), the total charge on the electrode is zero (shown in red) and that from the electrolyte is still negative (shown in blue). At the time when charging and discharging switch (at 100 ps), the charge from the in-pore electrolyte is less than that from the electrode. Moreover, the change in the charge from the in-pore electrolyte in the initial stage is slower than in the rest, because both cations and anions change at a lower rate in the initial stage of the charging and discharging process (Fig. 5a). The hysteresis in the negative electrode exhibits the same trend (Supplementary Fig. 16).

There is also a hysteresis in ion adsorption–desorption during charging and discharging in the open-electrode system (Supplementary Fig. 17), though weaker than in the nanoporous-electrode system. This hysteresis originates from the fact that the electrolyte ions do not respond to the electrode polarization in as timely a manner as the electrode charges (Supplementary Fig. 18a). This hysteresis could account for the negative potentials occurring at the initial stage of the charging and at the end of the discharging at the positive electrode (Fig. 2e and Extended Data Fig. 1). Details of the hysteresis and its contribution to the negative potential are provided in Supplementary Section 8 and Supplementary Figs. 17 and 18. Similarly, the negative value of potentials on the positive electrode of nanoporous supercapacitors can also be understood by the hysteresis of ion adsorption–desorption resulting in the negative charge from the electrolyte at the beginning of charging or the end of discharging (Fig. 5b).

## Discussion

The GCD-CPM method developed here can not only model the charging and discharging process of supercapacitors on applying electric currents of any form, but can also be combined with the previous CPM in potentiostatic mode to investigate complex charging modes at the molecular level, thereby helping to gain deep insights into the dynamics mechanisms and optimal charge–discharge modes. The dependence of capacitance on pore size has been intensively investigated by molecular simulations in the potentiostatic mode^3,37–39^. Such a dependence of charging dynamics, especially in the galvanostatic mode, is of significance and can be investigated by GCD-CPM. This method could also be used for other applications involving the dynamic formation and release of double layers, such as capacitive deionization^40^, batteries^41^, superlubricity^42^ and electrolyte gating^43^. Moreover, our work provides guidance for selecting molecular simulation methods for given simulation systems.

It is worthwhile noting that the position of the electrode is set as fixed in our modeling, which works for electrode systems with negligible electrode deformation during charging and discharging. Future directions in the modeling development will include the consideration of electrode deformation, which is essential for electrode materials experiencing large deformations, such as MXenes^44,45^.

## Methods

### GCD-CPM for modeling galvanostatic charge–discharge

We considered a classical electrochemical system containing *N* immobile electrode atoms (including *N*_pos_ positive electrode atoms and *N*_neg_ negative electrode atoms) and *M* mobile electrolyte atoms. The CPM considers Gaussian charge distributions for electrode atoms and point charges for electrolyte atoms^7,8^. The fluctuating charges on electrodes, with magnitude **q**, are extra degrees of freedom to keep the electrodes equipotential. The electrostatic energy of the system, *U*_ele_, is a function of atom positions **X** and electrode charge magnitude **q**, written as^8,14,46^

2 $$U_{\rm{ele}}\left( {{{{\mathbf{X}}}},{{{\mathbf{q}}}}} \right) = \frac{{{{{\mathbf{q}}}}^{{{\intercal}}}{{{{{A}{\mathbf{q}}}}}}}}{2} - {{{\mathbf{q}}}}^{{{\intercal}}}{{{\mathbf{B}}}}\left( {{{\mathbf{X}}}} \right) + C\left( {{{\mathbf{X}}}} \right).$$

The first and second terms in the right-hand side of equation (2) represent the electrode–electrode and electrode–electrolyte interactions, respectively, and the last term *C*(**X**) describes the electrolyte–electrolyte interaction. Matrix *A* with size *N* × *N*, depending on the position of electrode atoms, is defined as^8,14,46^

3 $$\begin{array}{l}{{{A}}}_{{{{ij}}}} = \frac{{8\uppi }}{V}\mathop {\sum }\limits_{{{{k}}}_1 \ge 0} \mathop {\sum }\limits_{{{{k}}}_2,{{{k}}}_3} \frac{{\cos \left( {{{{\mathbf{k}}}} \cdot {{{\mathbf{X}}}}_{{{{ij}}}}} \right)}}{{{{{k}}}^2}}{\rm{e}}^{ - \frac{{k^2}}{{4\alpha ^2}}} + \frac{{{{{\mathrm{erfc}}}}\left( {\alpha \left| {{{{\mathbf{X}}}}_{ij}} \right|} \right) - {{{\mathrm{erfc}}}}\left( {\frac{\eta }{{\sqrt 2 }}\left| {{{{\mathbf{X}}}}_{ij}} \right|} \right)}}{{\left| {{{{\mathbf{X}}}}_{ij}} \right|}}\\\qquad + 2\delta _{ij}\left( {\frac{\eta }{{\sqrt {2\uppi } }} - \frac{\alpha }{{\sqrt \uppi }}} \right),\end{array}$$

where *α* and *η* are the parameters of the Gaussian distribution, **k** is a reciprocal lattice vector, **X**_*ij*_ is the vector from electrode atom *i* to *j*, *V* is the volume of the simulation box, and erfc and *δ*_*ij*_ are the complementary error function and Dirac delta function, respectively. Vector **B** in length *N* is defined as

4 $${B}_i = - \frac{{8\uppi }}{V}\mathop {\sum }\limits_{{{{k}}}_1 \ge 0} \mathop {\sum }\limits_{{{{k}}}_2,{{{k}}}_3} \frac{{{\Re} \left[ {{\rm{e}}^{i{{{\mathbf{k}}}} \cdot {{{\mathbf{X}}}}_i}{{{{S}}}}^ \ast ({{{\mathbf{k}}}})} \right]}}{{{{{k}}}^2}}{\rm{e}}^{ - \frac{{{{{k}}}^2}}{{4\alpha ^2}}} - \mathop {\sum }\limits_j q_j\frac{{{{{\mathrm{erfc}}}}\left( {\alpha \left| {{{{\mathbf{X}}}}_{ij}} \right|} \right) - {{{\mathrm{erfc}}}}\left( {\eta \left| {{{{\mathbf{X}}}}_{ij}} \right|} \right)}}{{\left| {{{{\mathbf{X}}}}_{ij}} \right|}},$$

where *S*(**k**) is the structure factor of the electrolyte, **X**_*ij*_ is the vector from electrode atom *i* to electrolyte atom *j*, and *q*_*j*_ is the charge of electrolyte atom *j*. The potential on each electrode atom is $\frac{{\partial U_{\rm{ele}}}}{{\partial q_i}}$ (*i* for the electrode atom index).

To model the galvanostatic charge–discharge, the total electrode charge is constrained as $Q\left( t \right) = \mathop {\sum}\nolimits_{i = 1}^{N_{\rm{pos}}} {q_i}$ and $\mathop {\sum}\nolimits_{i = N_{\rm{pos}} + 1}^{N_{\rm{pos}} + N_{\rm{neg}}} {q_i} = - Q(t)$, where *q*_*i*_ is the charge of electrode atom *i*. In this work, *Q*(*t*) is a triangular wave for GCD due to the applied square-wave current (Fig. 1d). To keep the electrode equipotential at any time step and meet the constraints of the total charge of the electrode, we need to minimize the energy

5 $$U_t\left( {{{{\mathbf{X}}}},{{{\mathbf{q}}}}} \right) = U_{\rm{ele}} - {{{\mathbf{q}}}}^{{{\intercal}}}{\bf{\upphi}} + Q\left( t \right)\phi _{\rm{pos}} - Q\left( t \right)\phi _{\rm{neg}}.$$

The vector ${\bf{\upphi}} ^{{{\intercal}}} = [\phi _{\rm{pos}}{{{\mathbf{I}}}}_{{{{\rm{pos}}}}},\phi _{\rm{neg}}{{{\mathbf{I}}}}_{{{{\rm{neg}}}}}]$ is for the potentials on all electrode atoms, where $\phi _{\rm{pos}}\,(\phi _{\rm{neg}})$ is the potential on the positive (negative) electrode. ${{{\mathbf{I}}}}_{{{{\rm{pos}}}}}\,\,({{{\mathbf{I}}}}_{{{{\rm{neg}}}}})$ is a vector of size 1 × *N*_pos_ (1 × *N*_neg_), the elements of which are all one. Similar to the previous CPM simulations with step-like potentials, we solve a set of linear equations to minimize *U*_*t*_ at each time step, then obtain *ϕ*_pos_, *ϕ*_neg_ and electrode atom charges **q**. Mathematically, we have

6 $${{{{M}\mathbf{x}}}} = {{{\mathbf{n}}}}.$$

The size of matrix $${{{{M}}}} = \left[ {\begin{array}{*{20}{c}} {{{{A}}}} & {{{{E}}}} \\ {{{{D}}}} & 0 \end{array}} \right]$$ is (*N* + 2) × (*N* + 2), where $${{{{D}}}} = \left[ {\begin{array}{*{20}{c}} {{{{\mathbf{I}}}}_{{{{\rm{pos}}}}}} & 0 \\ 0 & {{{{\mathbf{I}}}}_{{{{\rm{neg}}}}}} \end{array}} \right]$$ and $${{{{E}}}} = \left[ {\begin{array}{*{20}{c}} { - {{{\mathbf{I}}}}_{{{{\rm{pos}}}}}^{{{\intercal}}}} & 0 \\ 0 & { - {{{\mathbf{I}}}}_{{{{\rm{neg}}}}}^{{{\intercal}}}} \end{array}} \right]$$. Unknowns of the linear equations are ${{{\mathbf{x}}}}^{{{\intercal}}} = \left[ {{{{\mathbf{q}}}}^{{{\intercal}}},\phi _{\rm{pos}},\phi _{\rm{neg}}} \right]$, and ${{{\mathbf{n}}}}^{{{\intercal}}} = \left[ { - {{{\mathbf{B}}}}^{{{\intercal}}},Q\left( t \right), - Q(t)} \right]$.

### Molecular dynamics simulations

For the supercapacitor with open electrodes, each electrode is modeled by three layers of graphene sheets (Fig. 1a). The simulation box has a volume of 4.254 × 4.176 × 48 nm^3^, with a distance of 10 nm between the innermost layers of the positive and negative electrodes. For the supercapacitor with nanoporous electrodes, each electrode is modeled as a slit-shaped pore with walls composed of three layers of graphene sheets (Fig. 1b). The pore size of 0.67 nm is defined as the accessible gap between the two innermost slit sheets in contact with the electrolyte, and the pore length is 8 nm. The dimensions of the simulation box are 4.1 nm, 3 nm and 32 nm. The simulation box of all systems is sufficiently large to ensure that the central region of the electrolyte reservoir is in a bulk-like state. The Lennard–Jones model of the carbon atom^47^ is taken for the electrode, and a coarse-grained model (Fig. 1b), which provides accurate thermodynamic and dynamic properties, is adopted for the ionic liquid, [EMIM][BF_4_] (ref. ^48^).

All simulations are performed in the NVT ensemble using customized MD software, GROMACS^49^ (see the ‘Code availability’ section for more information), with a time step of 2 fs and a Nosé–Hoover thermostat^50^ at a temperature of 400 K. The effects of the thermostat settings were explored (Supplementary Section 9 and Supplementary Figs. 19 and 20). The heat generation during GCD was calculated as the energy removed from the system to maintain its temperature^50,51^. To avoid energy drift and attain an accurate heat generation, our simulations are carried out in double precision^49^. A cutoff length of 1.2 nm is chosen to calculate non-electrostatic interactions and electrostatic interactions in real space. The electrostatic interactions in reciprocal space are computed using the particle mesh Ewald method^52^ with a fast Fourier transform grid spacing of 0.1 nm. Because the open-electrode system has a slab geometry, the three-dimensional Ewald summation with the correction term for the slab geometry is adopted in electrostatic interactions^5,53^.

For each simulation, the system is first run for 40 ns to reach equilibrium at the potential of zero surface charge. A square-wave current of 30 cycles is then applied on the supercapacitors (Fig. 1b). We average the data of the last 10 cycles to obtain results for a stable cycle, so the time mentioned above refers to the time relative to the beginning of the stable cycle. For the open-electrode system, the charge density of the positive electrode grows linearly from 0 to 9 μC cm^−2^ in the first half of each cycle and then decreases linearly to 0 in the second half cycle. The period of applied electric current is set as 50, 100 and 500 ps. For the nanoporous-electrode system, the surface charge density on the electrode is set to change linearly between ~0 and 4.96 μC cm^−2^, with periods of 200, 1,000 and 3,000 ps. For GCD-CPM, the charge on electrode atoms is obtained using the method mentioned above. For GCD-CCM, the potential at each electrode atom is computed as the sum of the potentials in the real space and reciprocal space. To ensure the accuracy of the simulation results, four to five independent simulations are run with different initial configurations for each system.

### Calculation of the probability distribution of the electrode atom charge and potential

For GCD-CPM, the probability distribution of electrode atom charges is *f*(*q*, *t*), defined as the ratio of *N*(*q*, *t*) to the total atoms of the electrode. *N*(*q*, *t*) refers to the number of electrode atoms with charges in the range of $q - {\rm{d}}q/2$ and $q + {\rm{d}}q/2$ at time *t*. We set d*q* as $\frac{{Q_{\rm{max}} - Q_{\rm{min}}}}{{N_{\rm{bin}}}}$, where *Q*_max_ and *Q*_min_ are the maximum and minimum charge values of all atoms at the electrode during the charging process, and *N*_bin_ is set as 300 here. Similarly, the probability distributions of electrode atom potentials in GCD-CCM can be obtained.

### Calculation of effective diffusion

The spatial and temporal distribution of charge densities inside the electrode pore, *ρ*_e_, is described by the charge diffusion equation (equation (1)). *D*_eff_ can be calculated by minimizing the difference in the in-pore charge density evolution between the molecular simulation and the diffusion equation. The initial and boundary conditions used to solve the minimization problem are taken directly from molecular simulations. Supplementary Figs. 7 and 8 show the evolution of in-pore charge density inside the electrode pore, obtained by GCD-CPM and GCD-CCM simulations and their fitting based on the minimization problem. Figure 3e and Supplementary Fig. 9 show the effective diffusion inside the positive and negative electrode pores obtained by solving the minimization problem.

### Dimensionless diffusion model for GCD

As already mentioned, the charge–discharge process of ionic liquids in a nanopore can be described by the charge diffusion equation (equation (1)) derived from mean-field theory^11,31^. For GCD, the initial and boundary conditions are set as

7a $$\left. {\rho _{\rm{e}}} \right|_{t = 0} = 0,$$

7b $$\left. {\frac{{\partial \rho _{\rm{e}}}}{{\partial z}}} \right|_{z = l} = 0,$$

7c $$\left. {\frac{{\partial \rho _{\rm{e}} }}{{\partial z}}} \right|_{z = 0} = - \frac{{I_0}}{{D_{\rm{eff}}}}S\left( {\frac{t}{P}} \right),$$

where *l* is the pore length and *S* is a square-wave function with period and amplitude of one. Equation (7a) indicates that the pore is not charged at *t* = 0. The boundary condition in equation (7b) is the zero normal flux at *z* = *l* (corresponds to the middle position of the open pore in our simulations), and the boundary in equation (7c) indicates an applied square-wave current with period *P* and amplitude *I*_0_, as shown in Fig. 1d.

As shown in Supplementary Fig. 21, the ions are distributed in the nanopore, and the electrode is equipotential. Taking the areal capacitance, *C*, along the nanopore as a constant, we divide the pore into numerous microcapacitors in parallel, with each having capacitance *C*d*z*. We then get

8 $$\rho _{\rm{e}}\left( {z,t} \right){\rm{d}}z = \left[ {\phi _1\left( {z,t} \right) - \phi _0\left( t \right)} \right]C{\rm{d}}z,$$

where *ϕ*_1_(*z*, *t*) is the potential in the electrolyte and *ϕ*_0_(*t*) the potential on the electrode. Using the notation for the potential drop from the electrolyte to electrode $\phi \left( {z,t} \right) = \phi _1(z,t) - \phi _0(t)$ and substituting equation (8) into equations (1) and (7), we respectively obtain the diffusion equation for the potential

9 $$\frac{{\partial \phi }}{{\partial t}} = D_{\rm{eff}}\frac{{\partial \phi ^2}}{{\partial ^2z}},$$

and initial and boundary conditions

10a $$\left. \phi \right|_{t = 0} = 0,$$

10b $$\left. {\frac{{\partial \phi }}{{\partial z}}} \right|_{z = l} = 0,$$

10c $$\left. {\frac{{\partial \phi }}{{\partial z}}} \right|_{z = 0} = - \frac{{I_0}}{{CD_{\rm{eff}}}}S\left( {\frac{t}{P}} \right).$$

It is worth noting that equations (9) and (10) are in the same form as the transmission line model^54,55^, because the charging process predicted by the transmission model also exhibits diffusive behavior^11^.

We define the dimensionless potential as ${{{\bar{{\varPhi}}}}} = \frac{{CD_{\rm{eff}}}}{{I_0l}}\phi$, dimensionless position as *Z* = *z*/*l*, dimensionless time as ${{{\bar{T}}}} = t/\tau$ ($\tau = l^2/D_{\rm{eff}}$ is the charging time constant) and the dimensionless period as *α* = *P*/*τ*. Hence, equation (9) can be written in dimensionless form as

11 $$\frac{{\partial {{{\bar{{\varPhi}}}}}}}{{\partial {{{\bar{T}}}}}} = \frac{{\partial ^2{{{\bar{{\varPhi}}}}}}}{{\partial Z^2}}.$$

The initial and boundary conditions become

12a $$\left. {{{{\bar{{\varPhi}}}}}} \right|_{{{{\bar{T}}}} = 0} = 0,$$

12b $$\left. {\frac{{\partial {{{{{\varPhi}}}}}}}{{\partial Z}}} \right|_{Z = 1} = 0,$$

12c $$\left. {\frac{{\partial {{{{{\varPhi}}}}}}}{{\partial Z}}} \right|_{Z = 0} = - S\left( {\frac{{{{{\bar{T}}}}}}{\alpha }} \right).$$

Accordingly, the potential difference between the positive and negative electrodes, ${{{\mathrm{{\Delta}}}\bar {\varPhi}}}({{{\bar{T}}}})$, could be obtained during the charge–discharge process, given by the GCD curve, as

13a $$\begin{array}{l}{{{{{\Delta}}}\bar {\varPhi}}}\left( {{{{\bar{T}}}}} \right) =\\\quad \frac{2}{3} + 2{{{\bar{T}}}} - \mathop {\sum }\limits_{n = 1}^\infty \frac{4}{{n^2\uppi ^2}}{\rm{e}}^{ - n^2\uppi ^2\bar T} + 4\mathop {\sum }\limits_{m = 1}^\infty \left( { - 1} \right)^m{{{{{\varPhi}}}}}_{\rm{step}}\left( {m,{{{\bar{T}}}}} \right),\end{array}$$

where

13b $$\begin{array}{l}{{{{{\varPhi}}}}}_{\rm{step}}\left( {m,{{{\bar{T}}}}} \right) =\\\quad \left[ {\frac{1}{3} + \left( {{{{\bar{T}}}} - \frac{{m\alpha }}{2}} \right)} \right]\theta \left( {{{{\bar{T}}}} - \frac{{m\alpha }}{2}} \right) - \mathop {\sum }\limits_{n = 1}^\infty \frac{2}{{n^2\uppi ^2}}{\rm{e}}^{ - \left( {{{{\bar{T}}}} - \frac{{m\alpha }}{2}} \right)n^2\uppi ^2}\theta \left( {{{{\bar{T}}}} - \frac{{m\alpha }}{2}} \right)\end{array}$$

and *θ* is the Heaviside step function. Therefore, the relation between ${{{{{\Delta}}}\bar {\varPhi}}}$ and ${{{\bar{T}}}}$, described by equation (13), reveals that the dimensionless GCD curve is only determined by the dimensionless period *α* (that is, the ratio of the electric current period to the charging time constant of the system).

### Materials for the experiment

Commercial activated-carbon cloth (ACC-5092-15) was purchased from Kynol, fabricated into the free-standing and binder-free electrode. Before use, it was dried at 120 °C under vacuum for 12 h. The ionic liquid [EMIM][BF_4_] (Iolitec Technologies) was purified at 85 °C by the Schlenk line for 24 h.

### Material characterization

Nitrogen sorption experiments were performed with an Autosorb iQ system (Quantachrome) at 77 K. The activated-carbon cloth was degassed at 10^−2^ Pa at a temperature of 200 °C for 10 h. The sample was found to exhibit a gravimetric Brunauer–Emmett–Teller surface area of 1,534 m^2^ g^−1^. The pore size analysis was calculated (Supplementary Fig. 11) using quenched-solid density functional theory, assuming a slit-shaped pore model. The average pore size of the micropores was calculated as ~0.72 nm on the basis of volume-weighted pore size *d*_50_.

### Assembly and instrumentation

Before fabrication, two 10-mm-diameter activated-carbon-cloth electrodes were soaked in a 10-ml glass vial filled with [EMIM][BF_4_] in an argon-filled glove box containing less than 0.01 ppm H_2_O and O_2_. A two-electrode electrochemical cell was then fabricated in the same glove box. After adding the electrolyte, the electrochemical cells were hermetically sealed and transferred out of the glove box. Electrochemical measurements were performed on a CS-350H workstation (Wuhan Corrtest Instrument Co.).

### Electrochemical measurements

For comparison with modeling, GCD tests were carried out for our cell. The setting times for charging and discharging were equal, which is not the regular setting with voltage limitation. A series of electric currents with different charge–discharge periods (~12–400 s) were used to find the same dimensionless period as for the simulation. Supplementary Fig. 12 shows the GCD curves obtained in the experiment, and their dimensionless periods are the same as those from the GCD-CPM simulations (their actual periods are 20, 260 and 360 s). To enable comparison with modeling, ohmic drops, mainly caused by the electrode resistance^23,32^ and not reflected in the simulations, were removed from the experimental GCD curves. The treated experimental curves were then compared with the GCD-CPM results using the dimensionless diffusion model, as shown in Supplementary Figs. 13 and 14. Areal capacitances extracted from GCD curves were obtained from^24^

14 $$C = 2\frac{{I{\Delta}t}}{{A{\Delta}V}},$$

where *C* is the areal capacitance of a single electrode, *I* is the constant current, Δ*t* is the discharge time, *A* is the surface area of the electrode and Δ*V* is the voltage change during discharge (the voltage change removes the ohmic drop from the experiments). The areal capacitances obtained by simulations and experiments are shown in Supplementary Fig. 15.

### Supplementary information

Supplementary Information Supplementary Figs. 1–20 and Discussion.

Peer Review Information

### Source data

Source Data Fig. 2 Source data for Fig. 2.

Source Data Fig. 3 Source data for Fig. 3.

Source Data Fig. 4 Source data for Fig. 4.

Source Data Fig. 5 Source data for Fig. 5.

Source Data Extended Data Fig. 1 Source data for Extended Data Fig. 1.

Source Data Extended Data Fig. 2 Source data for Extended Data Fig. 2.

## Data Availability

The simulation setting data for the galvanostatic charge–discharge of supercapacitors using the CPM and CCM approaches are available from Code Ocean^56^. The experimental data for the galvanostatic charge–discharge tests were generated on the laboratory electrochemical workstation with the settings described in the Methods section. Source data are provided with this paper.

## Code availability

The code for modeling the galvanostatic charge–discharge process in the molecular dynamics simulation using CPM and CCM approaches is available from Code Ocean^56^.
