# Supplementary material for: Modeling galvanostatic charge–discharge of nanoporous supercapacitors
Source: Nat Comput Sci. 2021 Nov 22;1(11):725–31. doi: 10.1038/s43588-021-00153-5 (PMC10766529; doi:10.1038/s43588-021-00153-5)
Supplement: Supplementary file 1 — Supplementary Figs. 1–20 and Discussion. [file 43588_2021_153_MOESM1_ESM.pdf]

Supplementary information

Modeling galvanostatic charge-discharge of nanoporous supercapacitors

Liang Zeng[1], Taizheng Wu[1], Ting Ye[1], Tangming Mo[1], Rui Qiao[2], and Guang Feng[1,*]

Table of Contents

Part 1. Stable cycle of charging-discharging process 2

Part 2. Potential and charge on open electrodes 3

Part 3. Comparison of EDL structures in open electrode systems 4

Part 4. Potential and charge on nanoporous electrodes 7

Part 5. Evolution of in-pore charge density and effective diffusion 8

Part 6. Comparison of heat generation of nanoporous systems 11

Part 7. Experimental result and validation of molecular modeling 12

Part 8. Hysteresis of ion adsorption-desorption 14

Part 9. Effect of thermostats on molecular simulations of GCD-CPM 17

Part 10. Sketch of the diffusion model 19

---

[1]State Key Laboratory of Coal Combustion, School of Energy and Power Engineering, Huazhong University of Science and Technology (HUST), Wuhan 430074, China. [2]Department of Mechanical Engineering, Virginia Tech, Blacksburg, VA 24061, USA. E-mail: gfeng@hust.edu.cn

Part 1. Stable cycle of charging-discharging process

We modeled the galvanostatic charging-discharging process of the supercapacitor for 30 cycles. After a few cycles, it will reach stability. Supplementary Fig. 1 shows that the number density of cations near the positive electrode, obtained from GCD-CPM simulations for an open electrode system with a period of 100 ps. In this work, all the data shown in the main text is in a stable cycle by averaging the data of the last ten cycles. Hence, the time mentioned in this work refers to the time relative to the beginning of the averaged stable cycle.

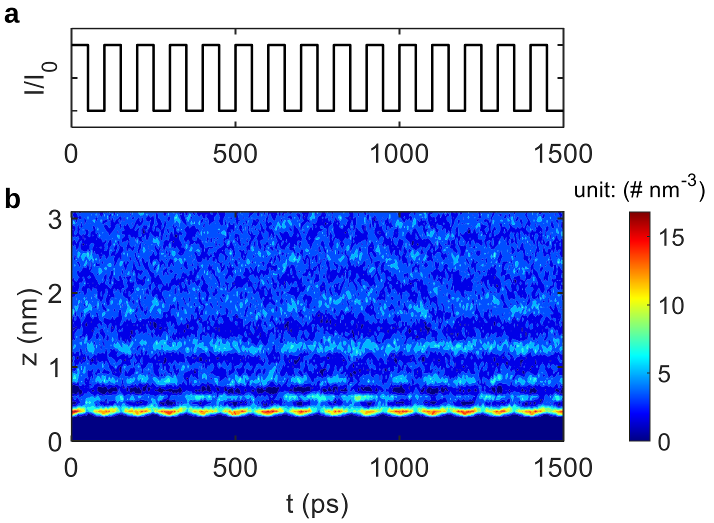

Supplementary Figure 1 | Input current and evolution of cation number density. a, Input current of galvanostatic charge-discharge. **b**, Time-evolution cation number density near positive electrodes, obtained from GCD-CPM simulations. Only the first 15 cycles are shown.

Part 2. Potential and charge on open electrodes

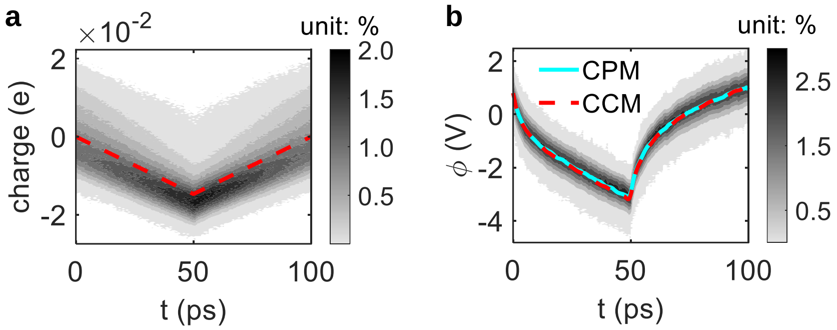

Supplementary Figure 2 | Charge and potential on electrode atoms in open electrode systems. **a,** Gray contour indicates probability distributions of negative electrode atom charges vary with time obtained from GCD-CPM simulations, and the dashed line is the average. **b**, Gray contour indicates the probability distributions of negative electrode atom potential vary with time obtained from GCD-CCM simulations. The red dashed line is the average. The solid cyan line is the potential of the negative electrode obtained from GCD-CPM simulations.

Part 3. Comparison of EDL structures in open electrode systems

As mentioned in the main text, the number densities of cations near the positive electrode by GCD-CPM and GCD-CCM are almost identical. For a more intuitive comparison, Supplementary Fig. 3 shows the number density of cations near the positive electrode at two moments (0 and 50 ps). Similarly, Supplementary Fig. 4 shows the number densities of cations near the negative electrode, anions near the positive electrode, and anions near the negative electrode. Supplementary Fig. 5 shows orientations of cations adsorbed on the negative electrode. As shown in Supplementary Fig. 5a, η is defined as the angle between the normal of the electrode surface and the normal of the cation plane, α is defined as the angle between the normal of the electrode surface and the vector pointing from the imidazole ring to ethyl, and β is defined as the angle between the normal of the electrode surface and vector pointing from imidazole ring to methyl. The first cation layer refers to the cations at the region between the electrode surface and the first valley of cation number density, as Supplementary Fig. 4 shows. One can find that the ion responses obtained by GCD-CPM and GCD-CCM are almost the same.

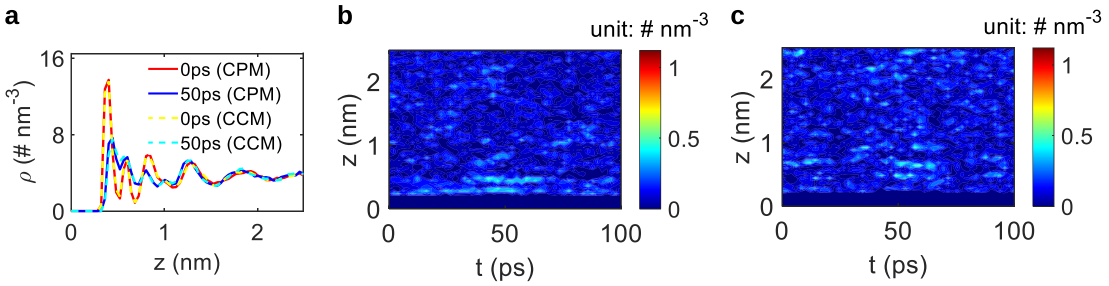

Supplementary Figure 3 | Comparison of the number density of cation near positive electrodes in open electrode systems. a, Number density at time 0 and 50 ps. **b-c**, Statistical error of time-evolution number density of cation obtained from GCD-CPM (**b**), and GCD-CCM (**c**).

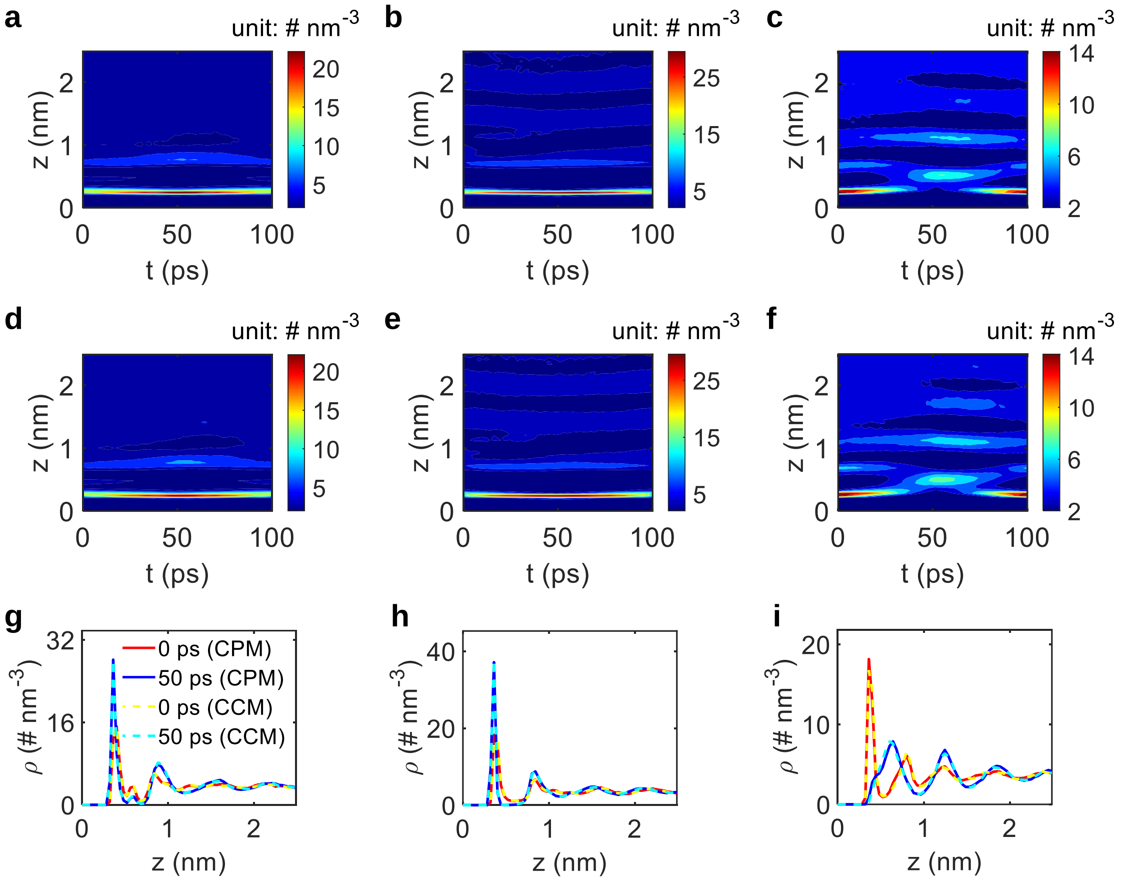

Supplementary Figure 4 | Ion number density in open electrode systems. a-c, Number density of cation near the negative electrode (**a**), anion near the positive electrode (**b**), and anion near the negative electrode (**c**) as a function of distance from electrode and time. The data was obtained from GCD-CPM simulations. **d-f**, Number density of cation near the negative electrode (**d**), anion near the positive electrode (**e**), and anion near the negative electrode(**f**) as a function of distance from electrode and time. The data was obtained from GCD-CCM simulations. **g-i**, Number density of cation near the negative electrode (**g**), anion near the positive electrode (**h**), and anion near the negative electrode (**i**) at time 0 and 50 ps.

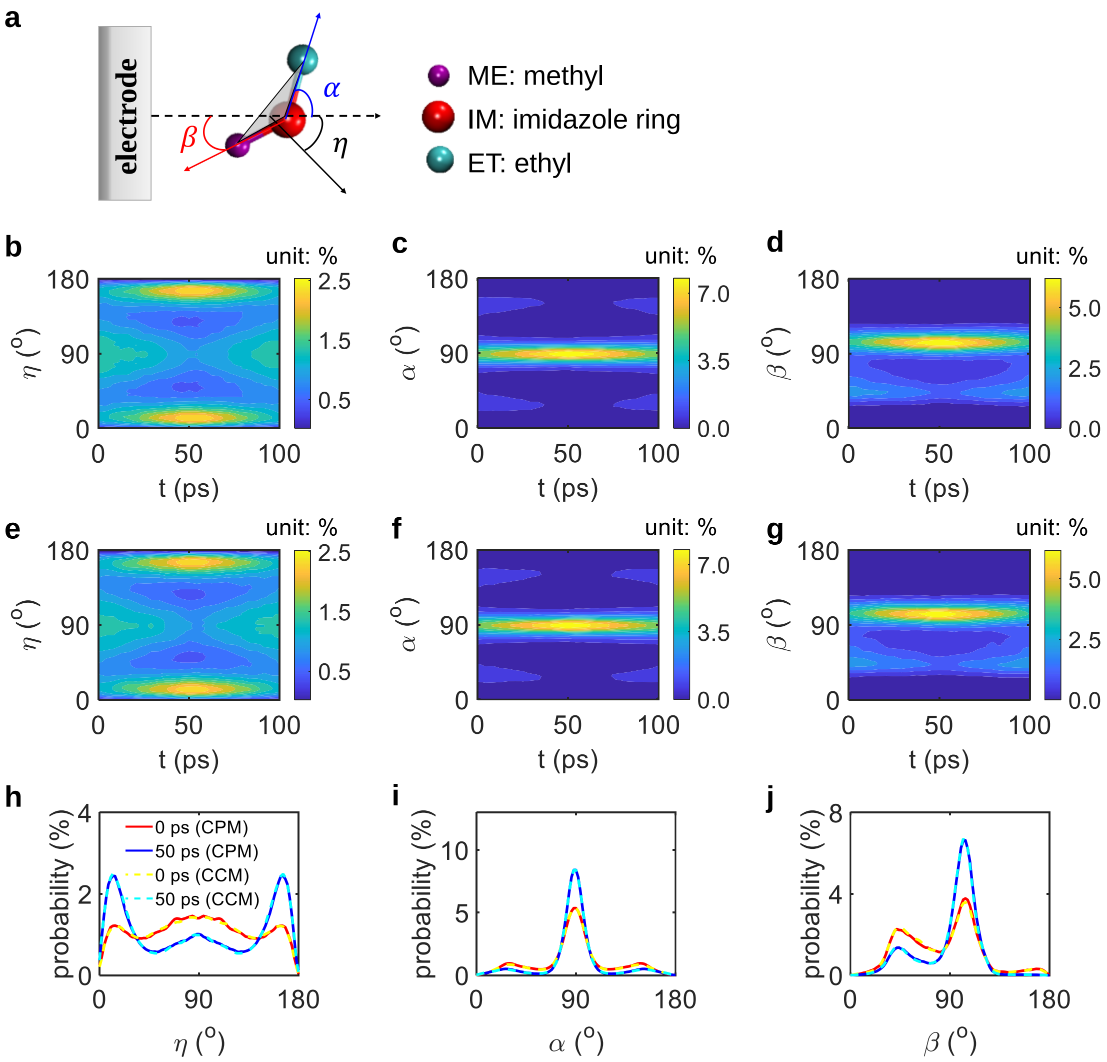

Supplementary Figure 5 | Orientation of the first cation layer adsorbed at the negative electrode of open electrode systems. a, Schematics for cation orientation. **b-d**, Probability distribution of η (**b**) α (**c**) and β (**d**) with time. The data was obtained from GCD-CPM simulations. **e-g**, Probability distribution of η (**e**) α (**f**) and β (**g**) with time. The data was obtained from GCD-CCM simulations. **g-i**, Probability distribution of η (**h**), α (**i**), and β (**j**) at 0 ps and 50 ps.

Part 4. Potential and charge on nanoporous electrodes

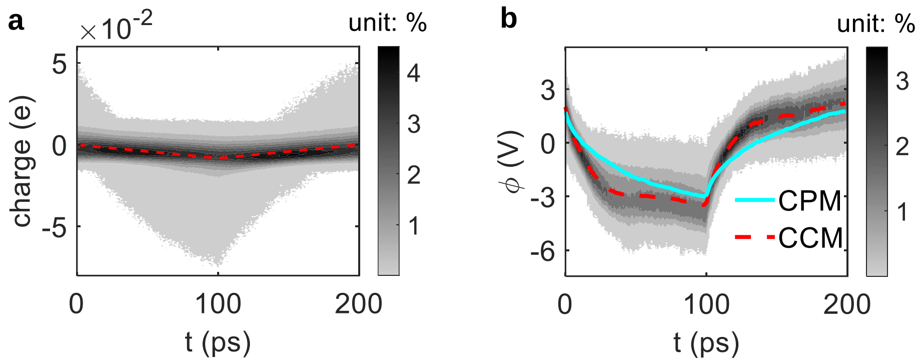

Supplementary Figure 6 | Charge and potential on electrode atoms in nanoporous electrode systems. a, Gray contour indicates probability distributions of negative electrode atom charges vary with time obtained from GCD-CPM simulations, and the dashed line is the average. **b**, Gray contour indicates the distributions of negative electrode atom potential vary with time obtained from GCD-CCM simulations, and the red dashed line is the average. The solid cyan line is the potential of the negative electrode obtained from GCD-CPM simulations.

Part 5. Evolution of in-pore charge density and effective diffusion

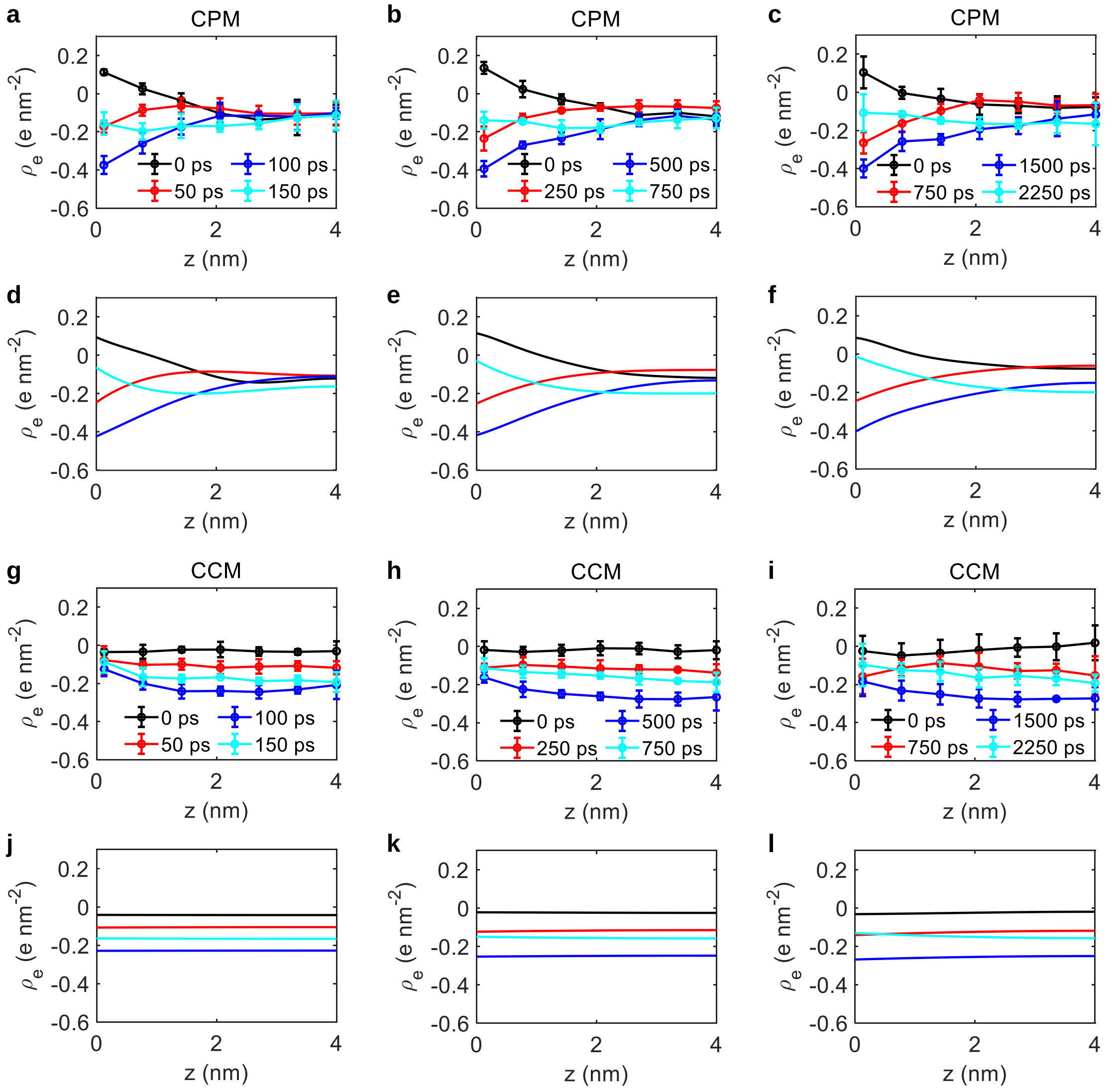

Supplementary Figure 7 | Evolution of in-pore charge density along the pore axis of the positive nanoporous electrode and corresponding fitting results. a-c, Evolution of in-pore charge density along the pore axis with the electric current period of 200 ps (**a**), 1000 ps (**b**), and 3000 ps (**c**) obtained from GCD-CPM simulations. **d-f**, The fitting in-pore charge density with the electric current period of 200 ps (**d**), 1000 ps (**e**), and 3000 ps (**f**), using GCD-CPM simulation data. **g-i**, Evolution of in-pore charge density along the pore axis with electric current periods of 200 ps (**g**), 1000 ps (**h**), and 3000 ps (**i**), obtained from GCD-CCM simulations. **j-l**, The fitting in-pore charge density with electric current periods of 200 ps (**j**), 1000 ps (**k**), and 3000 ps (**l**), using GCD-CCM simulation data. Error bars indicate one standard deviation of 4 independent simulations.

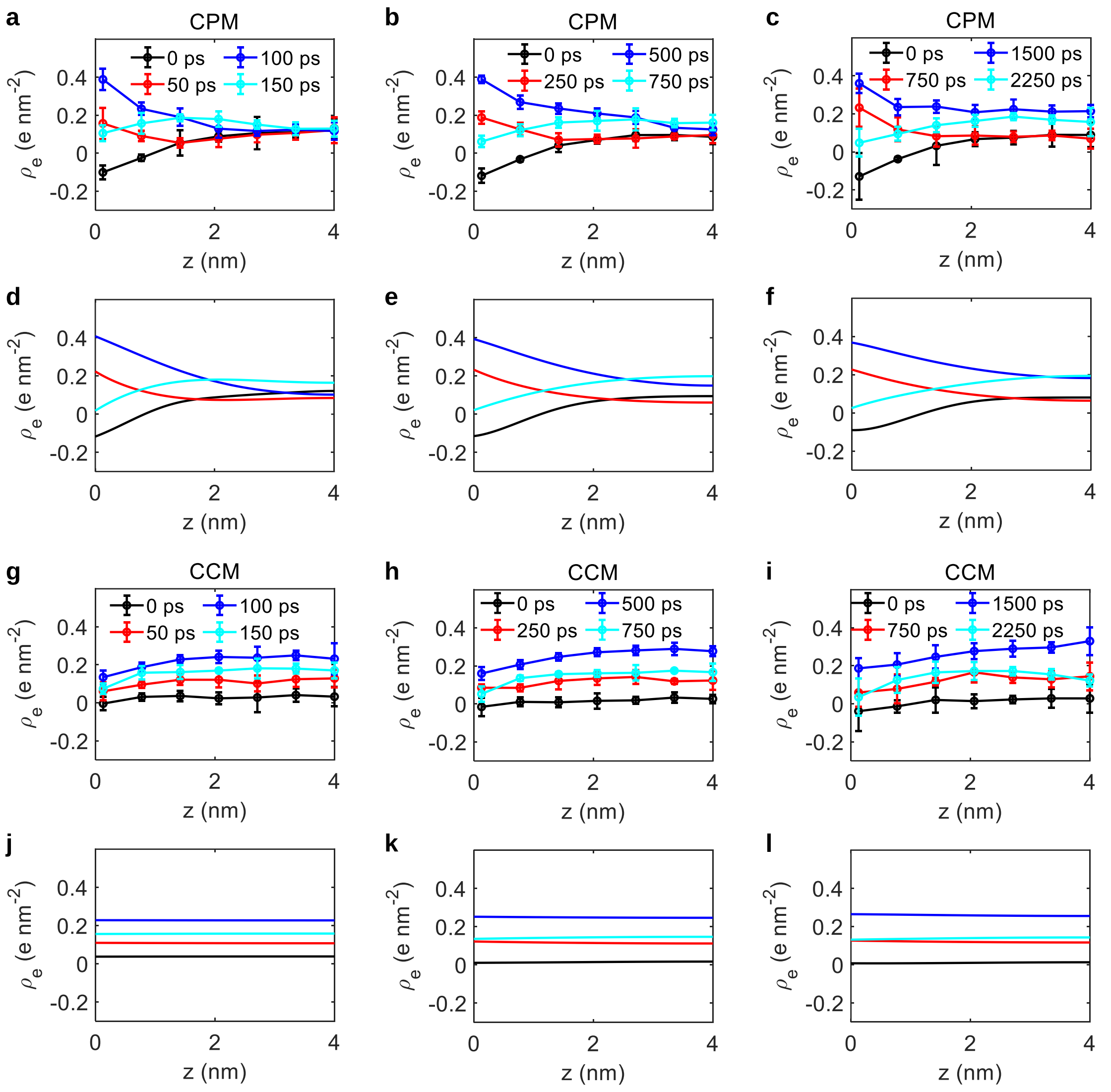

Supplementary Figure 8 | Evolution of in-pore charge density along the pore axis of the negative nanoporous electrode and corresponding fitting results. a-c, Evolution of in-pore charge density along the pore axis with the electric current period of 200 ps (**a**), 1000 ps (**b**), and 3000 ps (**c**) obtained from GCD-CPM simulations. **d-f**, The fitting in-pore charge density with the electric current period of 200 ps (**d**), 1000 ps (**e**), and 3000 ps (**f**), using GCD-CPM simulation data. **g-i**, Evolution of in-pore charge density along the pore axis of the electric current period of 200 ps (**g**), 1000 ps (**h**), and 3000 ps (**i**), obtained from GCD-CCM simulations. **j-l**, The fitting in-pore charge density with the electric current period of 200 ps (**j**), 1000 ps (**k**), and 3000 ps (**l**), using GCD-CCM simulation data. Error bars indicate one standard deviation of 4 independent simulations.

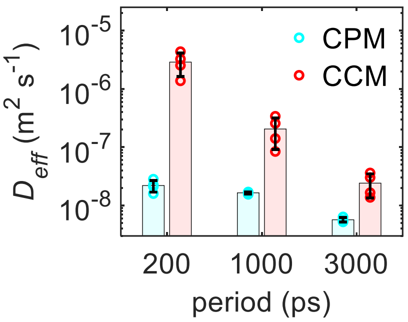

Supplementary Figure 9 | Effective diffusivity inside the negative electrode pore. Error bars indicate one standard deviation of 4 independent simulations.

Part 6. Comparison of heat generation of nanoporous systems

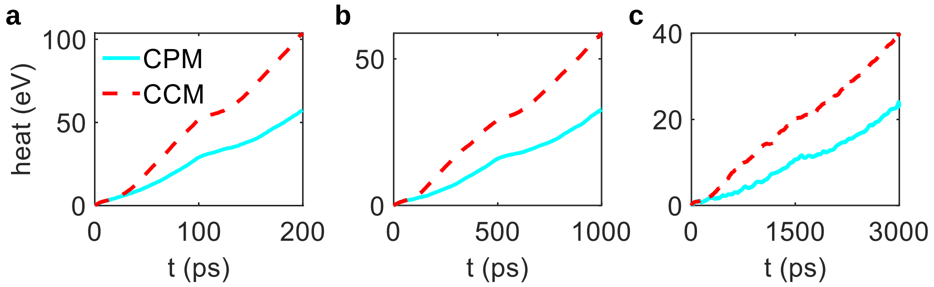

Supplementary Figure 10 | Evolution of heat generation in nanoporous electrode systems. **a-c,** Electric current periods are 200 ps (**a**), 1000 ps (**b**), and 3000 ps (**c**).

Part 7. Experimental result and validation of molecular modeling

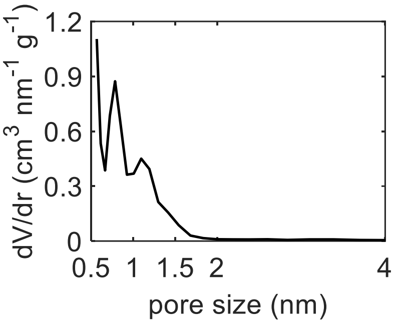

Supplementary Figure 11 | Pore size distribution of porous carbons. The carbon is ACC-5092-15, and the pore size is calculated by applying quenched-solid density functional theory (QSDFT), assuming a slit-shaped pore model.

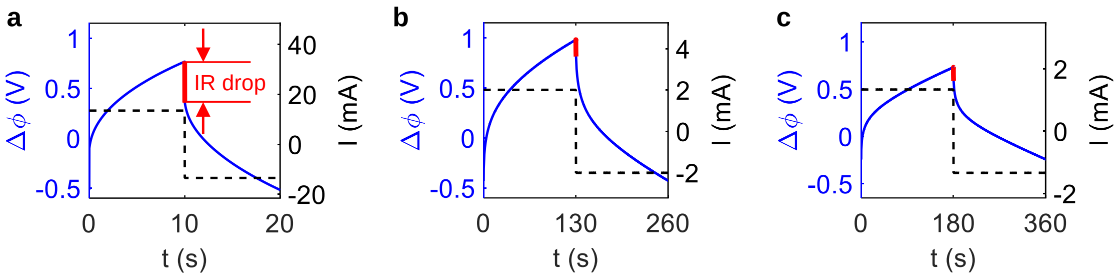

Supplementary Figure 12 | Experimental GCD curves. a-c, GCD curves obtained from experiments whose periods are 20 s (**a**), 260 s (**b**), and 360 s (**c**).

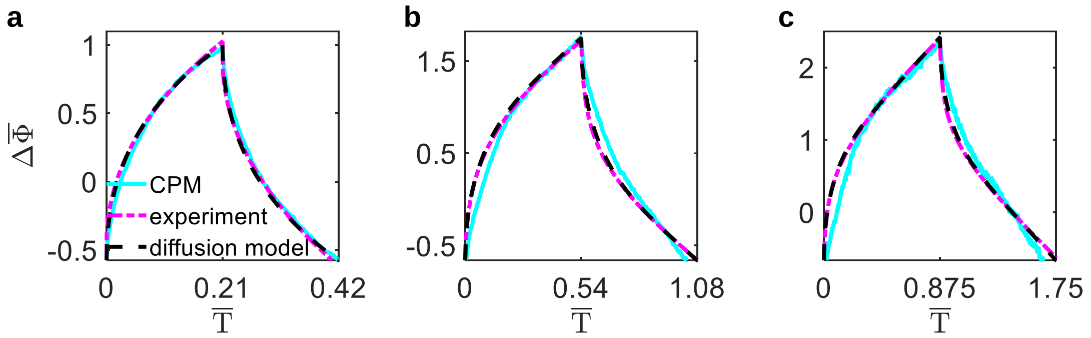

Supplementary Figure 13 | Validation of GCD-CPM by experiment. a-c, Dimensionless GCD curves obtained from GCD-CPM simulations and experiments, and their fitting by the diffusion model. The dimensionless periods are 0.42 (**a**), 1.08 (**b**), and 1.75 (**c**).

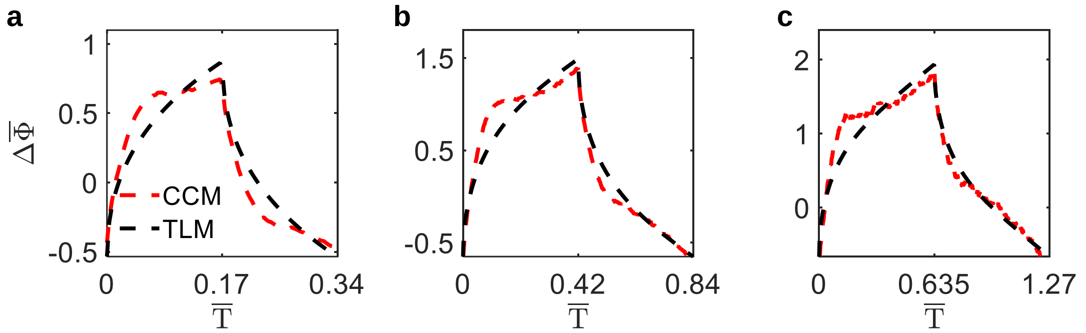

Supplementary Figure 14 | Fitting of GCD curves obtained from GCD-CCM simulations based on the dimensionless diffusion model. a-c, Dimensionless periods of 0.34 (**a**), 0.84 (**b**), and 1.27 (**c**).

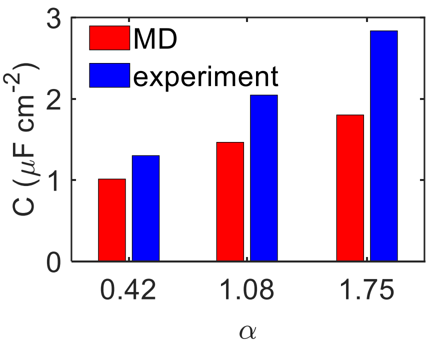

Supplementary Figure 15 | Areal capacitances extracted from GCD curves obtained by simulations and experiments.

Part 8. Hysteresis of ion adsorption-desorption

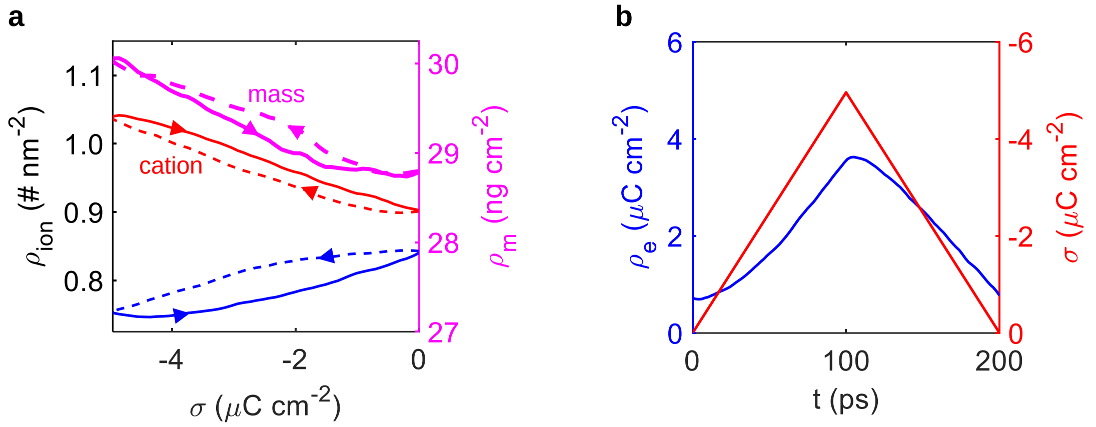

Supplementary Figure 16 | Hysteresis in the negative electrode of nanoporous electrode systems. a, Magenta line is the total mass density of in-pore electrolytes. Red and blue lines are the number densities of in-pore cations and anions, respectively. Solid (dashed) line is the discharging (charging) process. **b,** Blue line represents the charge density coming from in-pore ions, and red line is the charge density on the electrode. Results are for the negative electrode.

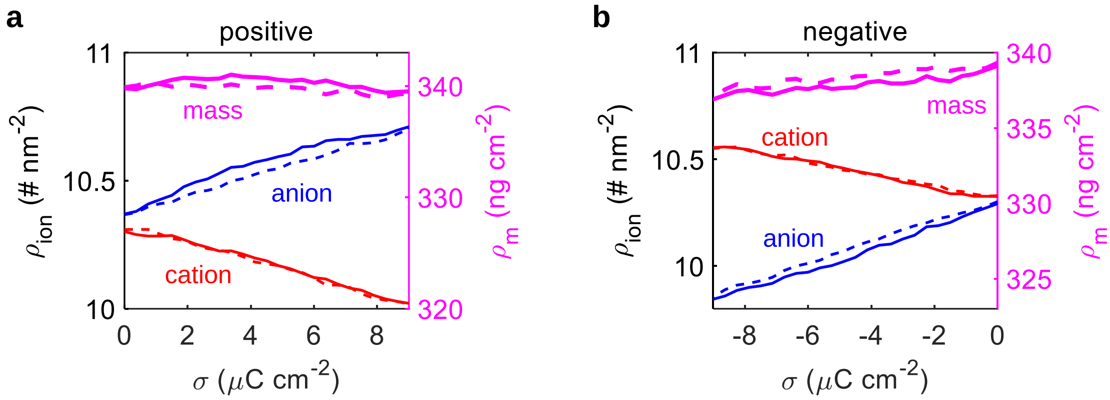

Supplementary Figure 17 | Hysteresis in open electrode systems. a-b, The average number density of cations and anions near the positive electrode (**a**) and the negative electrode (**b**). The data is from the region of 0–3 nm to the electrode surface. Magenta line is the total mass density from the electrolyte. Red and blue lines are the number densities of cations and anions, respectively. Solid (dashed) line is the discharging (charging) process. The electric current period is 100 ps.

Like the nanoporous electrode system, the charge from the electrolyte side changes slowly than that from the electrode side for open electrode systems. Here we explore the effect of hysteresis of ion adsorption-desorption on the GCD curves. σ_{lagging} is used to describe how the charge from the electrolyte lags behind that on the electrode, and it is defined as

$$\sigma_{\text{lagging}} = \sigma + \int_0^{L/2} \rho_e dz, \tag{S3}$$

where σ is the total surface charge density of one electrode and ρ_e is the charge density through the EDL near this electrode. L is the distance between the positive and negative electrodes.

For the open electrode systems, the potential difference between the positive and negative electrodes can be derived from charge distribution[1], as

$$\Delta\phi = \frac{\sigma}{\epsilon_0} L - \frac{1}{\epsilon_0} \int_0^L (L - z)\rho_e dz, \tag{S4}$$

where ϵ_0 is vacuum permittivity. We then divide the surface charge σ into two parts: the lagging charge σ_{lagging} and the balanced charge $\sigma - \sigma_{\text{lagging}}$. Then $\Delta\phi$ is divided consequently into

$$\Delta\phi_{\text{lagging}} = \frac{\sigma_{\text{lagging}}}{\epsilon_0} L, \tag{S5a}$$

$$\Delta\phi_{\text{rest}} = \frac{\sigma - \sigma_{\text{lagging}}}{\epsilon_0} L - \frac{1}{\epsilon_0} \int_0^L (L - z)\rho_e dz, \tag{S5b}$$

where $\Delta\phi_{\text{lagging}}$ is produced only by σ_{lagging}, and $\Delta\phi_{\text{rest}}$ is produced by $\sigma - \sigma_{\text{lagging}}$. The degree of charge lagging decreases as P increases (Supplementary Fig. 18a), since electrolyte ions require a longer time to respond to the electrode polarization. The asymmetry and the negative values of the GCD curve are caused by such a lagging charge, as shown in Supplementary Fig. 18b.

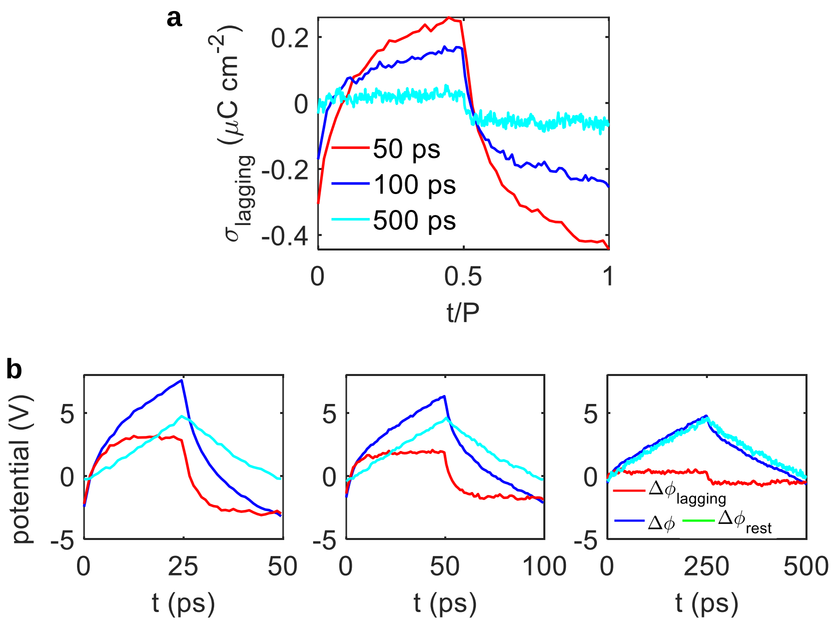

Supplementary Figure 18 | Charge lagging and its effect on GCD curves for open electrode systems. a, Evolution of lagging charge σ_{lagging}. **b,** Evolution of $\Delta\phi$, $\Delta\phi_{\text{lagging}}$, and $\Delta\phi_{\text{rest}}$.

Part 9. Effect of thermostats on molecular simulations of GCD-CPM

The common thermostat modes used in MD simulation include Nosé–Hoover[2], Berendsen[3], and V-rescaling[4] thermostats. To explore the effects of thermostat modes on the results, we took the same MD simulation systems with only changing Nosé–Hoover thermostat to Berendsen thermostat and V-rescaling thermostat in both open electrode system and nanoporous electrode system. MD results suggest that the GCD curves of the open electrode system (Supplementary Fig. 19a) and the nanoporous electrode system (Supplementary Fig. 19b) are independent of the thermostat modes. Since Nosé–Hoover thermostat can get the energy removed from the system to maintain the system at the target temperature[2, 5], which is the heat generation during galvanostatic charge-discharge in our work, the Nosé–Hoover thermostat was adopted in our work.

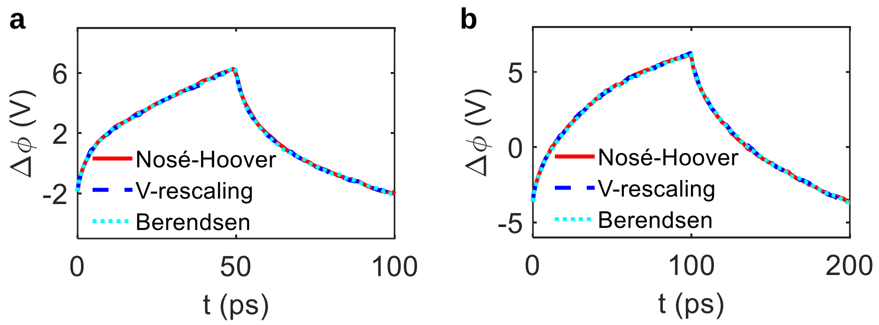

Supplementary Figure 19 | Effect of the thermostat mode on molecular simulations of GCD-CPM. **a-b**, GCD curve of the open electrode system with a current period of 100 ps (**a**) and the nanoporous electrode system with a current period of 200 ps (**b**).

Furthermore, the local temperature evolution in simulation systems using the Nosé–Hoover thermostat was monitored, where the temperature is calculated for different regions of a system[6, 7]. Herein, we divided an open electrode system into the reservoir region and EDL region (Supplementary Fig. 20a), and a nanoporous system into the in-pore region and reservoir region (Supplementary Fig. 20b). It can be found that the temperature difference between EDL and reservoir regions is very small for open electrode systems (Supplementary Fig. 20c). For the nanoporous electrode systems, the temperature differences between the in-pore region and reservoir region

are also small for systems with periods of 1000 ps and 3000 ps, but with a period of 200 ps, the temperature of the in-pore region is about 10 K higher than that of the reservoir (Supplementary Fig. 20d). Therefore, these new results suggest that the temperature gradient is very small for the open electrode system; however, there would be a temperature gradient for nanoporous electrode systems in some conditions.

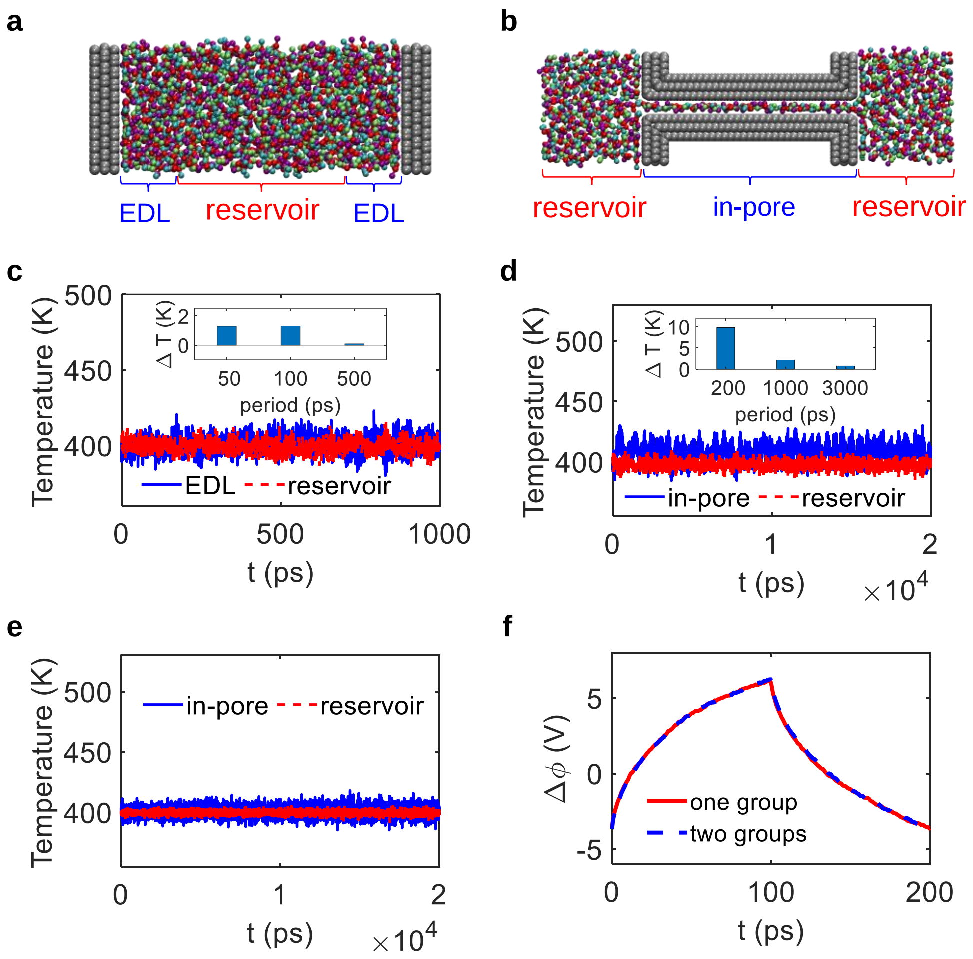

Supplementary Figure 20 | Effect of temperature gradient on molecular simulations of GCD-CPM. a-b, Sketch of open electrode system (**a**) and nanoporous electrode system (**b**) in which only half of the nanoporous electrode system is shown. **c-d**, Local temperature of the open electrode system (**c**), and nanoporous electrode system (**d**). The current period is 100 ps for the open electrode system and 200 ps for the nanoporous electrode system. The inset is the temperature difference between the region of EDL (in-pore) and the reservoir region. **e,** Local temperature of nanoporous electrode system where using thermostats with two groups. The current period is 200ps. **f,** Effect of temperature difference on the GCD curve in the nanoporous electrode system with a current period of 200 ps.

To explore the influence of such temperature differences on the results in our work, we set the Nosé–Hoover thermostat separately on the group of ions in the in-pore region and the group of ions in the reservoir region at the same target temperature of 400 K in the nanoporous system with a current period of 200 ps, compared with the previous setup that the thermostat controls the temperature of the overall electrolytes. By applying the Nosé–Hoover thermostat to the two groups separately, the temperature difference between the two regions becomes much smaller (from 10 K to around 1 K, see Supplementary Fig. 20e). We also obtain the GCD curve from MD simulations with the thermostat separately on two groups (see Supplementary Fig. 20f), and found that although the temperature gradient is changed by applying the Nosé–Hoover thermostat on different regions, the result is nearly the same as that with one thermostat group.

Thus, thermostat settings have negligible effects on MD simulations in our work.

Part 10. Sketch of the diffusion model

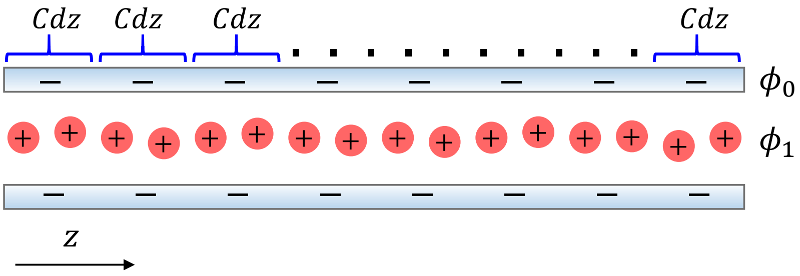

Supplementary Figure 21 | Sketch of the diffusion model for the charging of a nanopore filled with ionic liquids.

Supplementary References

1. Feng G., Zhang J. S., Qiao R. Microstructure and capacitance of the electrical double layers at the interface of ionic liquids and planar electrodes. *The Journal of Physical Chemistry C* 2009, 113(11)**:** 4549-4559.
2. Evans D. J., Holian B. L. The Nose–Hoover thermostat. *The Journal of Chemical Physics* 1985, 83(8)**:** 4069-4074.
3. Berendsen H. J. C. Transport properties computed by linear response through weak coupling to a bath. In: Meyer Madeleine, Pontikis Vassilis (eds). *Computer simulation in materials science: Interatomic potentials, simulation techniques and applications*. Springer Netherlands: Dordrecht, 1991pp 139-155.
4. Bussi G., Donadio D., Parrinello M. Canonical sampling through velocity rescaling. *The Journal of Chemical Physics* 2007, 126(1)**:** 014101.
5. Esposito M., Monnai T. Nonequilibrium thermodynamics and Nose−Hoover dynamics. *The Journal of Physical Chemistry B* 2011, 115(18)**:** 5144-5147.
6. Merlet C., Pean C., Rotenberg B., Madden P. A., Simon P., Salanne M. Simulating supercapacitors: Can we model electrodes as constant charge surfaces? *Journal of Physical Chemistry Letters* 2013, 4(2)**:** 264-268.
7. Pak A. J., Hwang G. S. Charging rate dependence of ion migration and stagnation in ionic-liquid-filled carbon nanopores. *The Journal of Physical Chemistry C* 2016, 120(43)**:** 24560-24567.
