# Supplementary material for: Modeling galvanostatic charge–discharge of nanoporous supercapacitors
Source: Nat Comput Sci. 2021 Nov 22;1(11):725–31. doi: 10.1038/s43588-021-00153-5 (PMC10766529; doi:10.1038/s43588-021-00153-5)
Supplement: Supplementary file 2 — Peer Review Information [file 43588_2021_153_MOESM2_ESM.pdf]

natureresearch

Peer Review Information

Journal: Nature Computational Science
Manuscript Title: Modeling galvanostatic charge-discharge of nanoporous supercapacitors
Corresponding author name(s): Guang Feng

Editorial Notes:

**Redactions – published data**	Parts of this Peer Review File have been redacted as indicated to remove third-party material.

Reviewer Comments & Decisions:

Decision Letter, initial version:

Dear Professor Feng,

Your manuscript "Modeling galvanostatic charge-discharge of nanoporous supercapacitors" has now been seen by 3 referees, whose comments are appended below. You will see that while they find your work of interest, they have raised points that need to be addressed before we can make a decision on publication.

The referees’ reports seem to be quite clear. Naturally, we will need you to address all of the points raised.

While we ask you to address all of the points raised, the following points need to be substantially worked on:

- Please provide statistical analysis and error estimation when presenting your results.
- Please provide clarifications on your comparisons, such as a further explanation on the validation of your results against TL model, since TL model itself presents limitations (as suggested by referee #3).
- Referee #1 raised a concern about the use of Nose-Hoover thermostat in your simulation. Please carefully address this technical concern.

- Referee #3 suggested to include effective properties which can be better compared with experimental values.
- Please discuss the correlation of the capacitance and pore size from the simulation (as suggested by referee #3).

Please use the following link to submit your revised manuscript and a point-by-point response to the referees' comments (which should be in a separate document to any cover letter):

[***REDACTED***]

** This url links to your confidential homepage and associated information about manuscripts you may have submitted or be reviewing for us. If you wish to forward this e-mail to co-authors, please delete this link to your homepage first. **

To aid in the review process, we would appreciate it if you could also provide a copy of your manuscript files that indicates your revisions by making of use of Track Changes or similar mark-up tools. Please also ensure that all correspondence is marked with your Nature Computational Science reference number in the subject line.

In addition, please make sure to upload a Word Document or LaTeX version of your text, to assist us in the editorial stage.

If you have any issues when updating your Code Ocean capsule during the revision process, please email the Code Ocean support team Cc'ing me.

To improve transparency in authorship, we request that all authors identified as 'corresponding author' on published papers create and link their Open Researcher and Contributor Identifier (ORCID) with their account on the Manuscript Tracking System (MTS), prior to acceptance. ORCID helps the scientific community achieve unambiguous attribution of all scholarly contributions. You can create and link your ORCID from the home page of the MTS by clicking on 'Modify my Springer Nature account'. For more information please visit please visit www.springernature.com/orcid.

We hope to receive your revised paper within three weeks. If you cannot send it within this time, please let us know.

We look forward to hearing from you soon.

Best regards,

natureresearch

Jie Pan, Ph.D.
Associate Editor
Nature Computational Science

Reviewers comments:

Reviewer #1 (Remarks to the Author):

The authors present a new method to adapt the Constant Potential Method for the simulation of electrode/electrolyte systems under galvanostatic charging conditions. The method is novel and would be of interest to scientists and engineers interested in modeling such systems for a variety of applications. Given the current high interest in research for the development of better batteries and supercapacitors, this method has the potential to become a standard tool and is thus suitable for Nature Computational Science. There are a few issues, however, that need to be addressed before publication.

1) The results are devoid of any analysis of statistical error. Simulation studies are statistical studies and all simulation results should be accompanied with error estimates, using, for example, block averaging. This needs to be addressed before publication is recommended.
2) Not all potential readers are necessarily familiar with the concepts of "galvanostatic" or "potentiostatic". I would suggest giving a short definition for clarity.
3) The simulations are performed using a Nose-Hoover thermostat to enforce NVT conditions. This can be problematic in driven systems such as this one where the Hamiltonian is time dependent (due the imposed current). Nose-Hoover only controls the average temperature and non-equilibrium conditions could lead to unintended temperature gradients in the system – the authors need to monitor the temperature profiles (gradients) within the system and discuss the degree to which the temperature profiles are non-uniform.
4) In the system with the planar "open" electrode system, is a 2-d or 3-d Ewald sum used? How does this choice affect the results?

Reviewer #2 (Remarks to the Author):

The manuscript by Zeng et al. presents an advanced methodology for atomistic modeling of super capacitors and demonstrates the importance to utilize this methodology for modeling of super capacitors with nanostructure electrodes. Specifically author demonstrate how to utilize constant

electrode potential approach in combination with galvanostatic mode of charging/discharging - i.e. the conditions the most closely reflecting experimental conditions. Authors compare the electric double layer structure, charge distributions, ion dynamics, etc. predicted using the new approach with corresponding predictions using a more simplified constant charge approach. For atomically flat, open structure electrodes the two methods predict very similar properties and hence application of the new advanced methodology is not essential. This outcome is expected as on atomically flat electrodes constant charge and constant potential approaches are know to give very similar results. However, on more realistic and higher capacitance nanostructure electrodes, the importance of application of the new approach is well illustrated in the manuscript by showing significantly different predictions for the two methods. The manuscript is very nicely organized, easy to read, and the figures in the manuscript and SI are well crafted to show complex correlations as a function of several variables. In my opinion the manuscript can be published in current form. The manuscript will make an important impact for the modeling community working in the energy storage (batteries, supercars, fuel cells) field where accurate modeling of electrode-electrolyte interfaces is challenging, yet badly needed for our understanding of complex electrochemical processes defining the performance of these energy storage devices.

Reviewer #3 (Remarks to the Author):

The article by Zeng et al. studies the impact of galvanostatic charge-discharge of nanoporous supercapacitors is explored through molecular dynamic simulations. The predictions are also validated through experiments while using a TL model as a bridge. Overall, the results shown in the article are interesting for the supercaps community, and the article is well-suited for Nature Computational Science. However, I do have several suggestions that the authors should address before the paper is ready for final publication.

1. My main concern is that the validation criteria appear to be a bit circular. The validation relies on the TL model, which have several limitations.
(a) The authors have used the TL model by de Levie, which was derived for thin double layer limit. Here, the diameter of the pore considered is 0.67 nm, and thus it is likely going to be comparable to the Debye length. The structure of the TL model remains the same for thick DLs (see ref. 41 that does solve thick DLs), but the quantitative values might change.
(b) These TL models, both de Levie and Gupta, have been developed for small potentials, which is not followed in experiments.
(c) Small note: Could authors elaborate a little bit more about the expected concentration of the ionic liquids (IL) they considered in molecular dynamics and experiments? The reason I ask is that this would be important to calculate the Debye length, which is eventually utilized in the TL model that the authors have utilized to validate their model with experiments.

Overall, I think the authors should acknowledge these limitations and perhaps soften their claims of validation because there are many assumptions in the TL model that are not true in experiments. I just feel that the claims are bit exaggerated when the authors say “dynamics are in excellent agreement”, when they rely on another model with its own limitations.

2. One of the interesting features that authors can calculate in their systems is the effective properties. While the effective diffusivities have been reported, could authors also extract a capacitance value? I think it would be very instructive to see the “absolute” capacitance value from MD simulations. For instance, it is well known that experimentally observed capacitance values are on the order of O(0.1) F/m^2 …It would we be instructive to know whether this is followed in the simulations reported here.

3. Building up on 2., could authors also comment on the effect of capacitance on pore size from their simulations? This is a hotly debated topic in the supercaps community where claims have suggested specific capacitance can decrease with increase in pore size and can also stay the same. Therefore, it would be good to know if the authors can show such dependence from their simulations.

Author Rebuttal to Initial comments

We have studied all the comments carefully and made a point-to-point response to each comment. Specifically, the reviewer’s comments are copied in blue, and each comment is followed by our response in black. The manuscript has been revised, accordingly, in red. After each response, we have provided what has been changed or added and where they are positioned in the revised main text and corresponding Supplementary Information.

To carefully respond to all the comments, we have accomplished a substantial amount of supplementary but necessary modeling work, performed the corresponding analysis, and incorporated the new work all in the revised version of the manuscript.

For your information, we briefly summarized the work done for this revision:

1) Statistical analysis and error estimate have been added to the results in the figures of the revised main text and Supplementary Information.

2) Based on a mean-field theory that describes the charging behavior of ionic liquids in sub-nanometer pores, a diffusion model was derived to compare the GCD results from simulations and experiments. Although the dimensionless form of the derived diffusion model has the same formation as the transmission line model, it can describe nanoporous systems in our work without the limitations of the classical TL model.

3) Based on additional MD simulations, the effects of thermostat settings (including thermostat mode and local temperature gradient) on the results in our work were found to be very minor.

4) The areal capacitances were extracted from the GCD curves obtained from MD

simulations and experiments. These capacitances match and are also consistent with capacitances measured by other groups.
5) The importance of the relationship between pore size and capacitance was discussed.
6) Other modifications include (1) clarification of the concepts “galvanostatic” and “potentiostatic”, (2) an explanation of Ewald summation for computing electrostatic interaction in open electrode systems with a slab geometry.

Responses to Reviewers’ Comments

Reviewer #1 (Remarks to the Author):

The authors present a new method to adapt the Constant Potential Method for the simulation of electrode/electrolyte systems under galvanostatic charging conditions. The method is novel and would be of interest to scientists and engineers interested in modeling such systems for a variety of applications. Given the current high interest in research for the development of better batteries and supercapacitors, this method has the potential to become a standard tool and is thus suitable for Nature Computational Science. There are a few issues, however, that need to be addressed before publication.

Response:
We thank the reviewer very much for carefully going through our work and giving constructive comments/suggestions to help us solidify our conclusions with more work and discussions.

1) The results are devoid of any analysis of statistical error. Simulation studies are statistical studies and all simulation results should be accompanied with error estimates, using, for example, block averaging. This needs to be addressed before publication is recommended.

Response:
We thank the reviewer for the useful comment, and then performed the statistical error analysis by calculating the standard deviation of the results of five independent simulation cases.

Figure R1.1 shows the evolutions of in-pore charge density (**Figure R1.1a-b**), effective diffusivity (**Figure R1.1c**) of in-pore electrolyte, and heat generation during a galvanostatic charging-discharging cycle (**Figure R1.1d**) in nanoporous electrode systems. The statistical errors of the data in this figure are represented by error bars, and the size of some error bars is similar to the marker size so that some error bars are covered by the markers (**Figure R1.1a-b**). Following this, for all data displayed in the

form of bars or lines with markers in our work, we added error bars to represent the statistical error in the revised manuscript and Supplementary Information.

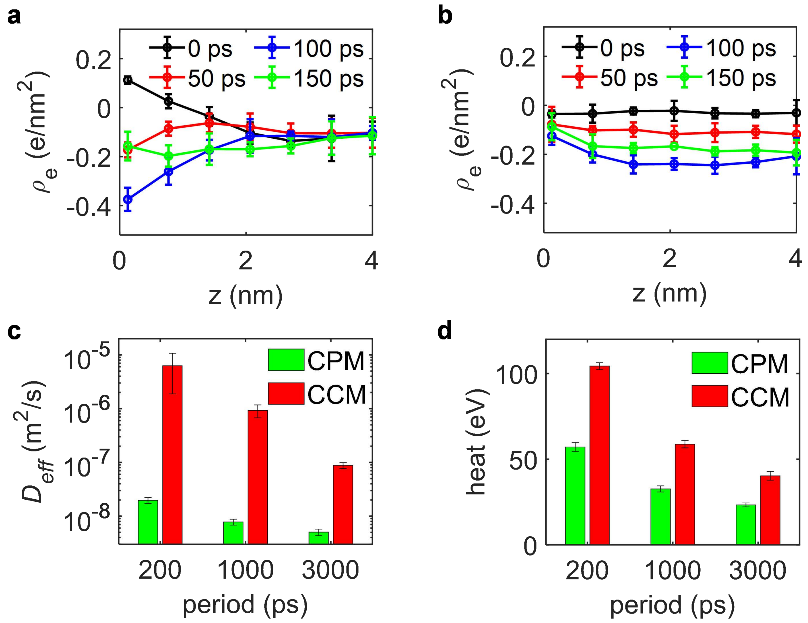

Figure R1.1 | GCD-CPM and GCD-CCM modeling of galvanostatic charge-discharge in nanoporous electrode systems. a-b, Evolution of in-pore charge density from electrolyte along the pore ($z = 0$ is the pore entrance), obtained from GCD-CPM simulations (**a**) and GCD-CCM simulations (**b**). **c,** Effective diffusivity of in-pore electrolyte under the positive polarization. **d,** Heat generation during a galvanostatic charging-discharging cycle. The error bar for each point is the standard deviation of the results based on five independent simulation cases.

Figure R1.2 shows the positive electrode potentials obtained by GCD-CPM and GCD-CCM in the open electrode system (**Figure R1.2a-b**) and nanoporous electrode system (**Figure R1.2c-d**), and their statistical errors are represented by the shaded regions. It can be seen that the regions of statistical error are similar to the width of the line, and thus we just add notes that the statistical error is comparable to the width of the line in the caption of the figures in the revised manuscript.

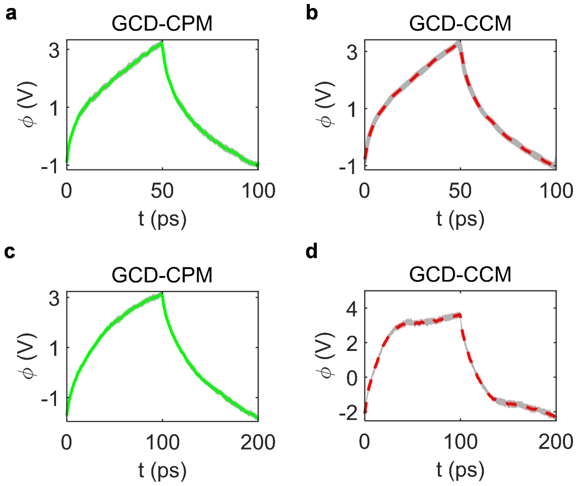

Figure R1.2 | Positive electrode potential for open electrode system by GCD-CPM (a) and GCD-CCM (b), and nanoporous electrode system by GCD-CPM (c) and GCD-CCM (d). Shaded regions indicate the range of statistical error.

Figure R1.3 shows evolutions of cation number density with time and distance from the positive electrode of open electrode system by GCD-CPM (**Figure R1.3a**) and GCD-CCM (**Figure R1.3c**), and their statistical error calculated from five independent simulation cases (**Figure R1.3b**, **Figure R1.3d**). The statistical error of the number density is small, and does not affect the position and magnitude of peaks in the number density profiles. Such statistical error will not change the key results and conclusions in our work, that is, the difference between the number density evolution of the open electrode system GCD-CPM and GCD-CCM is very small. Similarly, we calculated the orientations of the first cation layer absorbed at the negative electrode in the open electrode system with their statistical error as shown in **Figure R1.4**. The statistical error is also small and does not change the conclusion of our work. We added the figures of statistics error to the revised Supplementary Information.

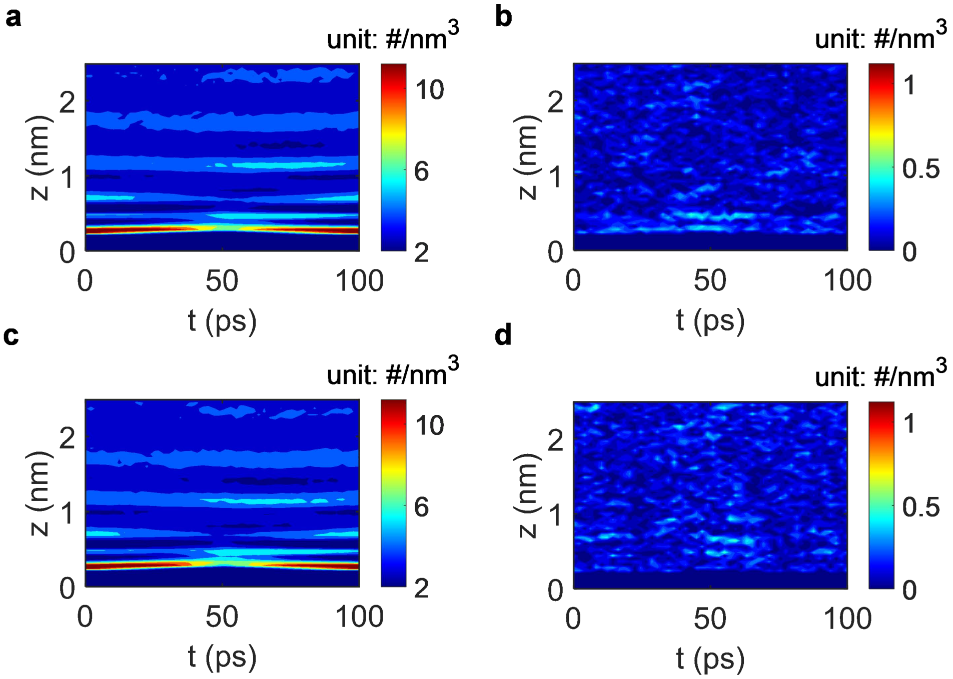

Figure R1.3 | Evolution of cation number density with time and distance from the positive electrode of open electrode systems. a-b, The data is obtained from GCD-CPM (a) with statistical error (b). c-d, The data is obtained from GCD-CCM (c) with statistical error (d).

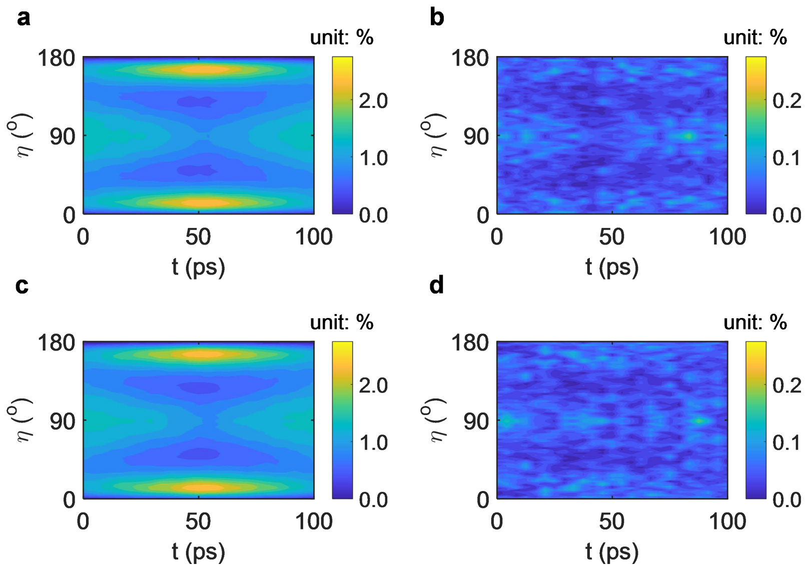

Figure R1.4 | Orientation of the first cation layer adsorbed at the negative electrode of open electrode systems. a-b, The data is obtained from GCD-CPM (**a**) with statistical error (**b**). **c-d,** The data is obtained from GCD-CCM (**c**) with statistical error (**d**). η is defined as the angle between the normal of the electrode surface and the normal of the cation plane.

Brief summary:

Based on the reviewer's suggestion, the above discussions and new data have been incorporated into the revised manuscript (Caption of **Fig. 2** of the main text, *Page* **5**, *lines* **86-94**; *Page* **5**, *lines* **102**). The figures with bars and lines with markers have been updated in the revised manuscript (**Fig. 3** of the main text, *Page* **6**, *lines* **119-129**; **Extended Data Fig. 2** of the main text, *Page* **23**, *lines* **574-579**) and Supplementary Information (**Supplementary Part 5** with **Supplementary Figure 7-9**, *Pages* **9-11**). Meanwhile, the statistical errors of evolutions of number density have been provided in the updated **Supplementary Part 3** with **Supplementary Figure 3** (*Page* **4**).

Specifically, the modification in the main text is:

"**Fig. 2 | Galvanostatic charge-discharge of open electrode systems. a-c,** GCD-CPM modeling: A snapshot of the positive electrode at 50 ps, with atoms colored by their instantaneous charges (**a**); Gray contour presents probability distributions of electrode charges varying with time, and the dashed line is the average (**b**); Evolution of ion number density with time and distance from the electrode (**c**). **d-f,** GCD-CCM modeling: A snapshot of the positive electrode at 50 ps, with atoms colored by their instantaneous potential (**d**); Gray contour indicates probability distributions of electrode potentials, and the red dashed line is the average (**e**); Evolution of cation number density (**f**). The solid green line in (**e**) is the electrode potential obtained by GCD-CPM. The electrode potential's error bar is close to the line width and thus not shown." (Caption of **Fig. 2** of the main text, *Pages* **5-6**, *lines* **86-94**)

"GCD-CCM is also found to be nearly the same as that by GCD-CPM (Fig. 2f vs. 2c, see Supplementary Fig. 3 for details)" (*Page* **5**, *lines* **102**)

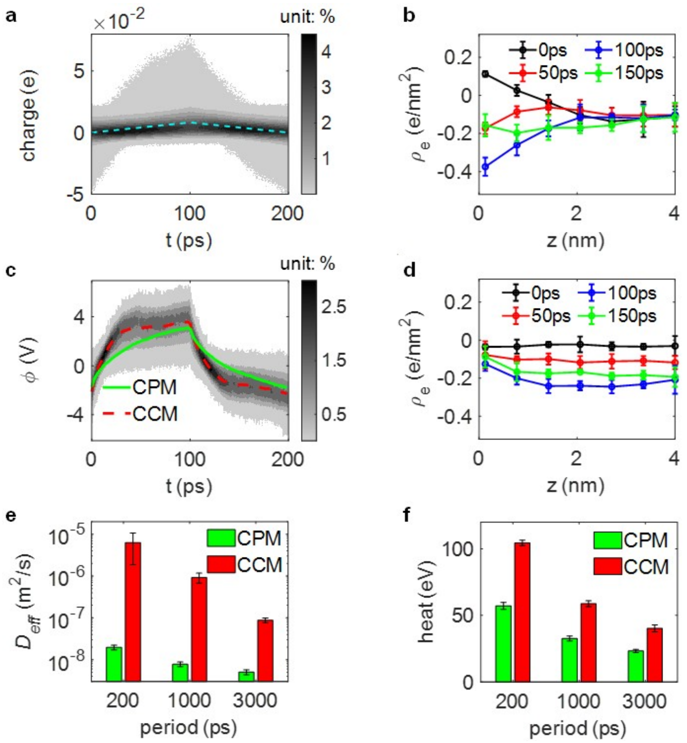

Fig. 3 | GCD-CPM and GCD-CCM modeling of galvanostatic charge-discharge in nanoporous electrode systems.

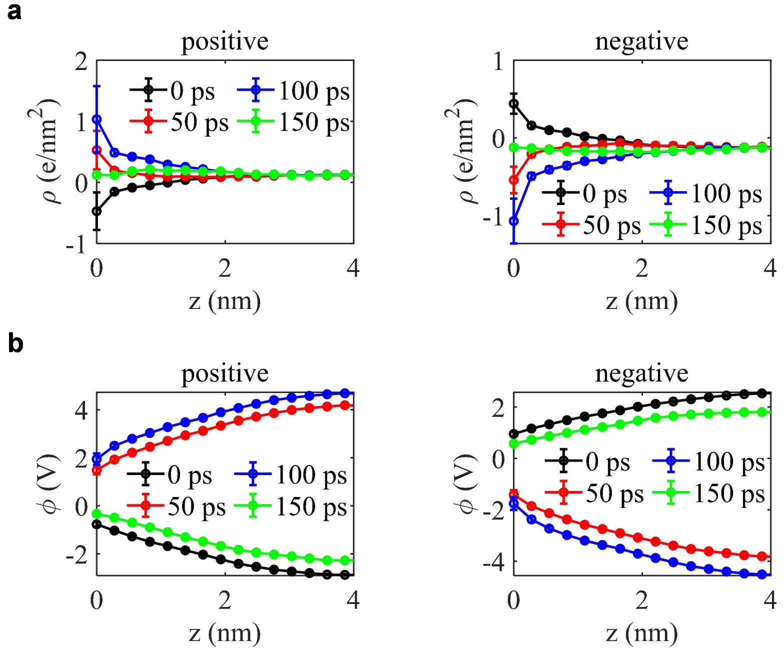

Extended Data Fig. 2 | Distribution of charge and potential on electrodes atoms in nanoporous electrode systems.

2) Not all potential readers are necessarily familiar with the concepts of "galvanostatic" or "potentiostatic". I would suggest giving a short definition for clarity.

Response:

We thank the reviewer for the useful comment since the words "galvanostatic" and "potentiostatic" may be unfamiliar to some potential readers. Following this suggestion, we clarified "potentiostatic" and "galvanostatic" by referring them to "electrodes are maintained at target voltages" and "electrodes to which given electric currents are applied", based on references 29 and 30.

With the above responses, we modified the main text as:

"Through this work, the potentiostatic mode refers to operations in which electrodes are maintained at target voltages, and the galvanostatic mode indicates that electrodes are applied with given electric currents[29, 30]." (**Introduction** section of the main text, *Page* **2**, *lines* **40-42**).

New references are added:

"29. Zhao F., Slade R. C. T., Varcoe J. R. Techniques for the study and development of microbial fuel cells: An electrochemical perspective. *Chemical Society Reviews* 2009, 38(7)**:** 1926-1939.

30. Kwabi D. G., Ji Y., Aziz M. J. Electrolyte lifetime in aqueous organic redox flow batteries: A critical review. *Chemical Reviews* 2020, 120(14)**:** 6467-6489."

3) The simulations are performed using a Nose-Hoover thermostat to enforce NVT conditions. This can be problematic in driven systems such as this one where the Hamiltonian is time dependent (due the imposed current). Nose-Hoover only controls the average temperature and non-equilibrium conditions could lead to unintended temperature gradients in the system – the authors need to monitor the temperature profiles (gradients) within the system and discuss the degree to which the temperature profiles are non-uniform.

Response:

We thank the reviewer for these insightful comments on the thermostat. The common thermostat modes used in MD simulation include Nosé–Hoover[1], Berendsen[2], and Vrescaling[3] thermostats. To explore the effects of thermostat modes on the results, we took the same MD simulation systems with **only changing** Nosé–Hoover thermostat to Berendsen thermostat and V-rescaling thermostat in both open electrode system and nanoporous electrode system. MD results suggest that the GCD curves of the open electrode system (**Figure R1.5a**) and the nanoporous electrode system (**Figure R1.5b**) are independent of the thermostat modes. Since Nosé–Hoover thermostat can get the energy removed from the system to maintain the system at the target temperature[1, 4],

which is the heat generation during galvanostatic charge-discharge in our work, the Nosé–Hoover thermostat was adopted in our work.

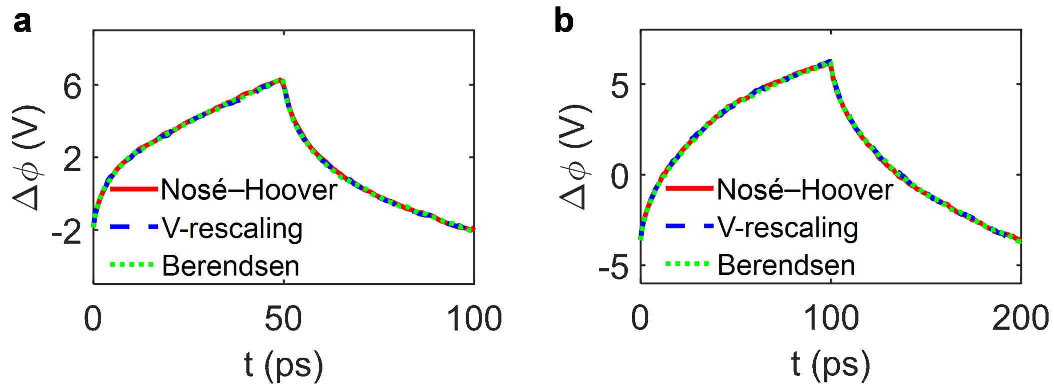

Figure R1.5 | Effect of the thermostat mode on molecular simulations of GCD-CPM. a-b, GCD curve of the open electrode system with a current period of 100 ps (**a**) and the nanoporous electrode system with a current period of 200 ps (**b**).

Furthermore, based on the reviewer's suggestion, the local temperature evolution in simulation systems using the Nosé–Hoover thermostat was monitored, where the temperature is calculated for different regions of a system[5, 6]. Herein, we divided an open electrode system into the reservoir region and EDL region (**Figure R1.6a**), and a nanoporous system into the in-pore region and reservoir region (**Figure R1.6b**). It can be found that the temperature difference between EDL and reservoir regions is very small for open electrode systems (**Figure R1.6c**). For the nanoporous electrode systems, the temperature differences between the in-pore region and reservoir region are also small for systems with periods of 1000 ps and 3000 ps, but with a period of 200 ps, the temperature of the in-pore region is about 10 K higher than that of the reservoir (**Figure R1.6d**). Therefore, these new results suggest that the temperature gradient is very small for the open electrode system; however, there would be a temperature gradient for nanoporous electrode systems in some conditions, which agrees with what the reviewer mentioned.

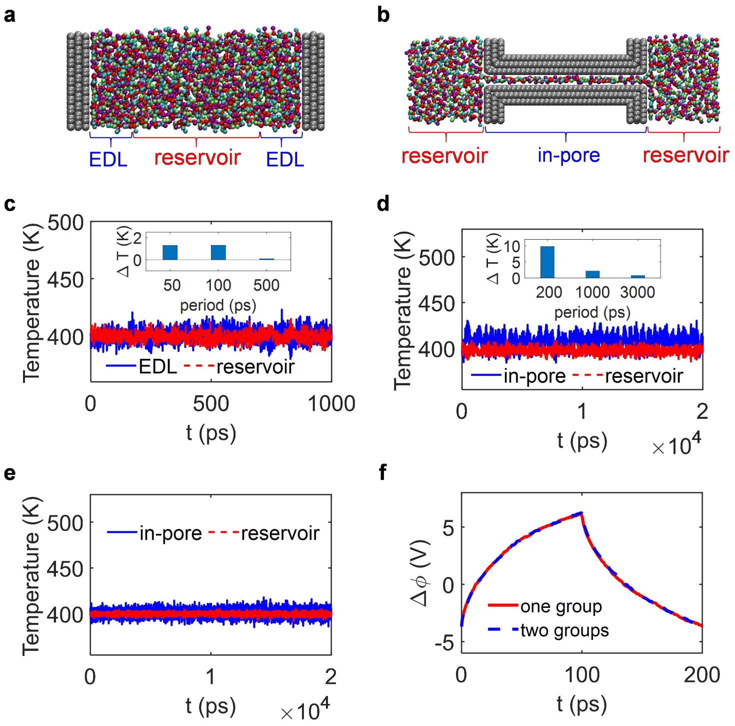

Figure R1.6 | Effect of temperature gradient on molecular simulations of GCD-CPM. **a-b**, Sketch of open electrode system (**a**) and nanoporous electrode system (**b**). Only half of the nanoporous electrode system is shown. **c-d**, Local temperature of the open electrode system (**c**), and nanoporous electrode system (**d**). The current period is 100 ps for the open electrode system and 200 ps for the nanoporous electrode system. The inset is the temperature difference between the region of EDL (in-pore) and the region of the reservoir under different current periods. **e,** Local temperature of nanoporous electrode system where using thermostats with two groups. The current period is 200ps. **f,** Effect of temperature difference on the GCD curve in the nanoporous electrode system with a current period of 200 ps.

To explore the influence of such temperature differences on the results in our work, we set the Nosé–Hoover thermostat separately on the group of ions in the in-pore region and the group of ions in the reservoir region at the same target temperature of 400 K in the nanoporous system with a current period of 200 ps, compared with the previous setup that the thermostat controls the temperature of the overall electrolytes. By applying the Nosé–Hoover thermostat to the two groups separately, the temperature difference between the two regions becomes much smaller (from 10 K to around 1 K, see **Figure R1.6e**). We also obtain the GCD curve from MD simulations with the thermostat separately on two groups (**Figure R1.6f**), and found that although the temperature gradient is changed by applying the Nosé–Hoover thermostat on different regions, the result is nearly the same as that with one thermostat group.
Thus, thermostat settings have negligible effects on MD simulations in our work.

Brief summary:
Based on the reviewer's suggestion, the above discussions and new data have been incorporated into the revised manuscript (**Methods** section, *Page* **14**, *lines* **311-313**), with a new part: **Supplementary Part 9** with **Supplementary Figure 19-20** (*Pages* **20-21**).
Specifically, the modification in the **Methods** section is:
"The effects of the thermostat settings were also explored, indicating that they can be negligible for simulations (Supplementary Part 9 with Supplementary Figs. 19-20)." (**Methods** section, *Page* **14**, *lines* **311-313**)
The modification in the Supplementary Information is:
"Part 9. Effect of thermostats on molecular simulations of GCD-CPM
The common thermostat modes used in MD simulation include Nosé–Hoover[8], Berendsen[9], and V-rescaling[10] thermostats. To explore the effects of thermostat modes on the results, we took the same MD simulation systems with only changing Nosé–Hoover thermostat to Berendsen thermostat and V-rescaling thermostat in both open electrode system and nanoporous electrode system. MD results suggest that the GCD curves of the open electrode system (Supplementary Fig. 19a) and the nanoporous electrode system (Supplementary Fig. 19b) are independent of the thermostat modes. Since Nosé–Hoover thermostat can get the energy removed from the system to maintain the system at the target temperature[8,11], which is the heat generation during galvanostatic charge-discharge in our work, the Nosé–Hoover thermostat was adopted in our work.

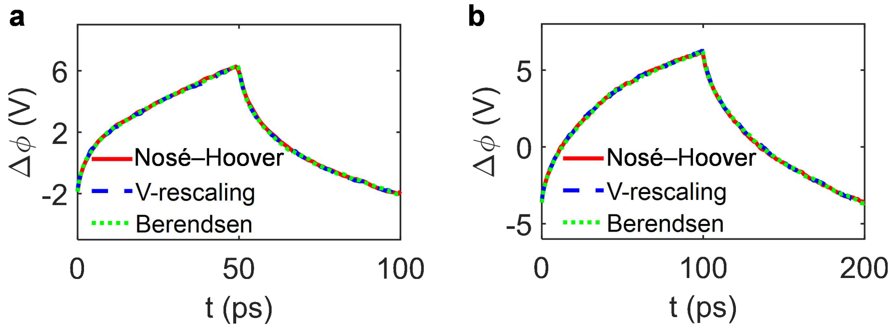

Supplementary Figure 19 | Effect of the thermostat mode on molecular simulations of GCD-CPM. **a-b**, GCD curve of the open electrode system with a current period of 100 ps (**a**) and the nanoporous electrode system with a current period of 200 ps (**b**).

Furthermore, the local temperature evolution in simulation systems using the Nosé–Hoover thermostat was monitored, where the temperature is calculated for different regions of a system[12, 13]. Herein, we divided an open electrode system into the

reservoir region and EDL region (Supplementary Fig. 20a), and a nanoporous system into the in-pore region and reservoir region (Supplementary Fig. 20b). It can be found that the temperature difference between EDL and reservoir regions is very small for open electrode systems (Supplementary Fig. 20c). For the nanoporous electrode systems, the temperature differences between the in-pore region and reservoir region are also small for systems with periods of 1000 ps and 3000 ps, but with a period of 200 ps, the temperature of the in-pore region is about 10 K higher than that of the reservoir (Supplementary Fig. 20d). Therefore, these new results suggest that the temperature gradient is very small for the open electrode system; however, there would be a temperature gradient for nanoporous electrode systems in some conditions.

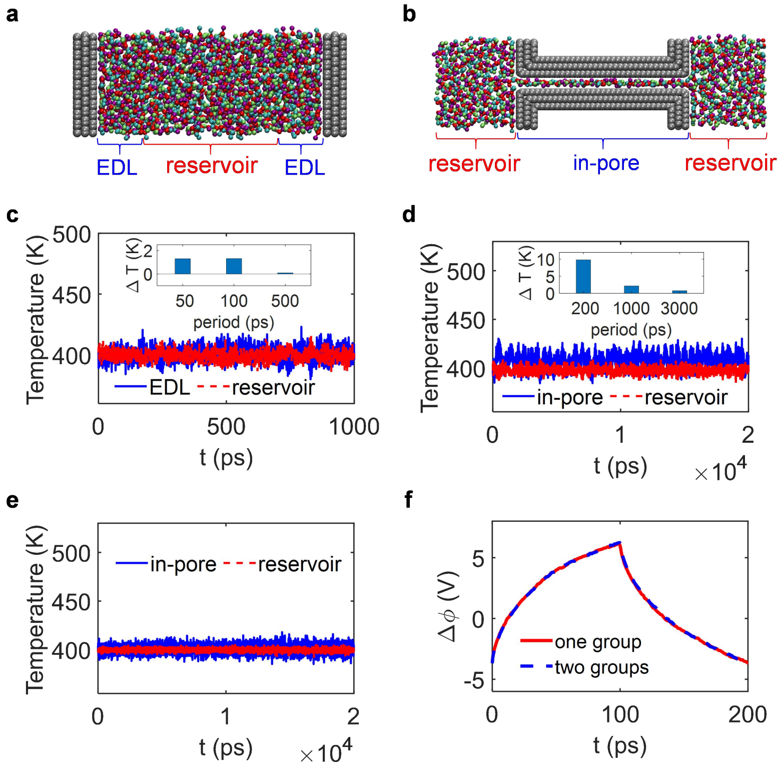

Supplementary Figure 20 | Effect of temperature gradient on molecular simulations of GCD-CPM. **a-b**, Sketch of open electrode system (**a**) and nanoporous electrode system (**b**) in which only half of the nanoporous electrode system is shown. **c-d**, Local temperature of the open electrode system (**c**), and nanoporous electrode system (**d**). The current period is 100 ps for the open electrode system and 200 ps for the nanoporous electrode system. The inset is the temperature difference between the region of EDL (in-pore) and the reservoir region. **e,** Local temperature of nanoporous electrode system where using thermostats with two groups. The current period is 200ps. **f,** Effect of temperature difference on the GCD curve in the nanoporous electrode system with a current period of 200 ps.

To explore the influence of such temperature differences on the results in our work, we set the Nosé–Hoover thermostat separately on the group of ions in the in-pore region and the group of ions in the reservoir region at the same target temperature of 400 K in the nanoporous system with a current period of 200 ps, compared with the previous setup that the thermostat controls the temperature of the overall electrolytes. By applying the Nosé–Hoover thermostat to the two groups separately, the temperature difference between the two regions becomes much smaller (from 10 K to around 1 K, see Supplementary Fig. 20e). We also obtain the GCD curve from MD simulations with the thermostat separately on two groups (see Supplementary Fig. 20f), and found that although the temperature gradient is changed by applying the Nosé–Hoover thermostat on different regions, the result is nearly the same as that with one thermostat group. Thus, thermostat settings have negligible effects on MD simulations in our work. (**Part 9**, *Pages* **19-21**)

4) In the system with the planar "open" electrode system, is a 2-d or 3-d Ewald sum used? How does this choice affect the results?

Response:

We appreciate the reviewer for carefully reading and commenting on the technique of electrostatic interaction calculation in a channel-type simulation system. The open electrode systems with a slab geometry here have no periodicity in the normal direction of the electrodes but periodicity in two dimensions parallel to the electrode surface, so that the 3-d Ewald summation cannot be used anymore. In our work, the method used to calculate electrostatic interaction in open electrode systems is the 3-d Ewald summation (here it is particle mesh Ewald[7]) **with a correction term for the slab geometry,** which could achieve the same result as the 2-d Ewald summation but takes less computational time[8, 9]. This method has been shown to well model the open surface systems with electric double layers[9-12].

Based on the reviewer's suggestion, the above discussion has been incorporated into the revised manuscript (**Methods** section, *Page* **14**, *lines* **319-321**):

"Since the open electrode system has a slab geometry, the three-dimensional Ewald summation with the correction term for the slab geometry is adopted in electrostatic interactions[5, 63-66]."

New references are added:

"63. Yeh I.-C., Berkowitz M. L. Ewald summation for systems with slab geometry. *The Journal of Chemical Physics* 1999, 111(7): 3155-3162.
64. Fedorov M. V., Kornyshev A. A. Ionic liquid near a charged wall: Structure and capacitance of electrical double layer. *The Journal of Physical Chemistry B* 2008, 112(38): 11868-11872.
65. Zhan C., Zhang Y., Cummings P. T., Jiang D. Computational insight into the capacitive

performance of graphene edge planes. *Carbon* 2017, 116: 278-285.
66. Zhang Y., Cummings P. T. Effects of solvent concentration on the performance of ionicliquid/ carbon supercapacitors. *ACS Applied Materials & Interfaces* 2019, 11(45): 42680-42689.

Reviewer #2 (Remarks to the Author):

The manuscript by Zeng et al. presents an advanced methodology for atomistic modeling of super capacitors and demonstrates the importance to utilize this methodology for modeling of super capacitors with nanostructure electrodes. Specifically authors demonstrate how to utilize constant electrode potential approach in combination with galvanostatic mode of charging/discharging - i.e. the conditions the most closely reflecting experimental conditions. Authors compare the electric double layer structure, charge distributions, ion dynamics, etc. predicted using the new approach with corresponding predictions using a more simplified constant charge approach. For atomically flat, open structure electrodes the two methods predict very similar properties and hence application of the new advanced methodology is not essential. This outcome is expected as on atomically flat electrodes constant charge and constant potential approaches are known to give very similar results. However, on more realistic and higher capacitance nanostructure electrodes, the importance of application of the new approach is well illustrated in the manuscript by showing significantly different predictions for the two methods. The manuscript is very nicely organized, easy to read, and the figures in the manuscript and SI are well crafted to show complex correlations as a function of several variables. In my opinion the manuscript can be published in current form. The manuscript will make an important impact for the modeling community working in the energy storage (batteries, supercars, fuel cells) field where accurate modeling of electrode-electrolyte interfaces is challenging, yet badly needed for our understanding of complex electrochemical processes defining the performance of these energy storage devices.

Response:

We appreciate the reviewer very much for the positive evaluation of our work with the key summary. We hope our revision would further solidify such evaluations that "The manuscript will make an important impact for the modeling community working in the energy storage (batteries, supercars, fuel cells) field where accurate modeling of electrode-electrolyte interfaces is challenging".

Reviewer #3 (Remarks to the Author):

The article by Zeng et al. studies the impact of galvanostatic charge-discharge of nanoporous supercapacitors is explored through molecular dynamic simulations. The predictions are also validated through experiments while using a TL model as a bridge. Overall, the results shown in the article are interesting for the supercaps community,

and the article is well-suited for Nature Computational Science. However, I do have several suggestions that the authors should address before the paper is ready for final publication.

Response:
We appreciate the reviewer for carefully assessing our work and giving quite constructive comments/suggestions directing more work and discussions needed to clarify our conclusions.

1. My main concern is that the validation criteria appear to be a bit circular. The validation relies on the TL model, which have several limitations.
(a) The authors have used the TL model by de Levie, which was derived for thin double layer limit. Here, the diameter of the pore considered is 0.67 nm, and thus it is likely going to be comparable to the Debye length. The structure of the TL model remains the same for thick DLs (see ref. 41 that does solve thick DLs), but the quantitative values might change.
(b) These TL models, both de Levie and Gupta, have been developed for small potentials, which is not followed in experiments.
(c) Small note: Could authors elaborate a little bit more about the expected concentration of the ionic liquids (IL) they considered in molecular dynamics and experiments? The reason I ask is that this would be important to calculate the Debye length, which is eventually utilized in the TL model that the authors have utilized to validate their model with experiments.
Overall, I think the authors should acknowledge these limitations and perhaps soften their claims of validation because there are many assumptions in the TL model that are not true in experiments. I just feel that the claims are bit exaggerated when the authors say "dynamics are in excellent agreement", when they rely on another model with its own limitations.

Response:
We thank the reviewer very much for these insightful comments on the TL model. We agree with the reviewer that the original TL model by de Levie[13] does have limitations, since it was developed for situations where the thickness of electric double layers (characterized by the Debye length) is much less than the pore size, the solution is dilute, and the applied voltage is mild. Further developed by Gupta[14], the TL model can be used to describe the charging behavior in nanopores when the electric double layers overlap, although there is still a limitation for high voltage. However, the TL model has been successfully adopted as an **empirical model** for charging nanopores beyond the limitations of the TL model[15-20]. For example, it can be used to fit well the charging behavior from MD simulations of nanoporous electrodes and *solvent-free ionic liquids* under high voltages (1~4 V).[15-17] In the experiment, the TL model is also taken to

interpret and fit the impedance spectroscopy data of supercapacitors composed of nanoporous electrodes and electrolytes that are not dilute solutions (*e.g.*, 1 M [TEA][TFB]/ACN[18], 30 wt% KOH aqueous solution[20], ionic liquid [EMIM][TFSI][19]).

Recent theoretical and computational studies have provided new insights into the apparent success of the TL model. The essence of the classical TL model is that charging is governed by a diffusion-type equation (for a given electrode-electrolyte setup, the diffusion coefficient can be fitted from experimental/computational data). For charging sub-nanometer pores filled with solvent-free ionic liquids, a mean-field theory has shown that the charging is a diffusive process, and its kinetics can be described by a diffusion equation[21, 22], which has the same *form* as the TL model. Specifically, a diffusion-type equation can describe the charging of ionic liquids in nanopores as discussed in Ref. 21: "*For instance, in single nanopores, the classical transmission line model is valid only when the pore size is much larger than the double-layer thickness (in some cases it may capture the shape of charging curves correctly also in the case of extremely narrow pores--this is, however, rather by 'coincidence' as we shall see)*." and "*Interestingly, the diffusion-type equation arises also in the transmission line model (TLM). This may explain the success of the TLM in interpreting impedance spectroscopy data for nanoporous electrodes*."

To more rigorously describe our results in light of the above information, we no longer claim to use the TL model to link the simulation and experiments. Instead, we utilize the diffusion model derived from the charge diffusion equation. Below, we show how to derive the diffusion model for the galvanostatic charge-discharge process based on the charge diffusion equation from the mean-field theory[21, 22].

First, we start with the charge diffusion equation in Refs. 21, 22 to describe the charge density ρ_e of in-pore ions at time t and location along pore axis z, as

$$\frac{\partial \rho_e}{\partial t} = D_{eff}\frac{\partial \rho_e^2}{\partial^2 z}, \quad (1)$$

where D_{eff} is effective diffusivity. For the galvanostatic charge-discharge process, the initial and boundary conditions are set as:

$$\rho_e|_{t=0} = 0, \quad (2a)$$

$$\left.\frac{\partial \rho_e}{\partial z}\right|_{z=l} = 0, \quad (2b)$$

$$\left.\frac{\partial \rho_e}{\partial z}\right|_{z=0} = -\frac{I_0}{D_{eff}} S\left(\frac{t}{P}\right), \quad (2c)$$

where l is pore length, and S is a square-wave function whose period and amplitude are both one. Equation 2a indicates the pore is uncharged at $t = 0$. The boundary condition in Eq. 2b is the zero normal flux at $z = l$ (corresponds to the middle position

of the open pore in our simulations), and the boundary in Eq. 2c indicates the applied square-wave current with a period P and an amplitude I_0.

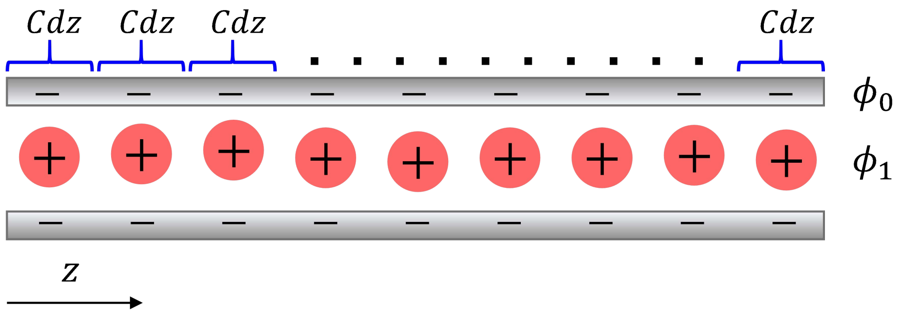

Figure R3.1 | Sketch of the diffusion model for the charging of a nanopore filled with ionic liquids.

As shown in **Figure R3.1**, the ions are distributed in the nanopore, and the electrode is equipotential. Taking the areal capacitance, C , along the nanopore as a constant, we divide the pore into numerous micro-capacitors in parallel with each having the capacitance of Cdz. Then we can get

$$\rho_e(z, t)dz = [\phi_1(z, t) - \phi_0(t)]Cdz, \tag{3}$$

where $\phi_1(z, t)$ is potential in the electrolyte and $\phi_0(t)$ is potential of electrode. Using the notation for the potential drop from the electrolyte to electrode $\phi(z, t) = \phi_1(z, t) - \phi_0(t)$ and substituting Eq. 3 into Eq. 1 and Eq. 2, we, respectively, obtain the diffusion equation for potential

$$\frac{\partial \phi}{\partial t} = D_{eff}\frac{\partial \phi^2}{\partial^2 z}, \tag{4}$$

and initial and boundary conditions

$$\phi|_{t=0} = 0, \tag{5a}$$

$$\left.\frac{\partial \phi}{\partial z}\right|_{z=l} = 0, \tag{5b}$$

$$\left.\frac{\partial \phi}{\partial z}\right|_{z=0} = -\frac{I_0}{CD_{eff}}S\left(\frac{t}{P}\right). \tag{5c}$$

We define the dimensionless potential $\overline{\Phi} = \frac{CD_{eff}}{I_0 l}\phi$, dimensionless position $Z = z/l$,

dimensionless time $\overline{\mathrm{T}} = t/\tau$ ($\tau = l^2/D_{eff}$ is the charging time constant), and the dimensionless period $\alpha = P/\tau$. Hence, Eq. 5 can be written in dimensionless form as

$$\frac{\partial\overline{\Phi}}{\partial\overline{\mathrm{T}}} = \frac{\partial^2\overline{\Phi}}{\partial Z^2}. \qquad (6)$$

The initial and boundary conditions become as

$$\overline{\Phi}|_{\overline{\mathrm{T}}=0} = 0, \qquad (7a)$$

$$\left.\frac{\partial\Phi}{\partial Z}\right|_{Z=1} = 0, \qquad (7b)$$

$$\left.\frac{\partial\Phi}{\partial Z}\right|_{Z=0} = -S\left(\frac{\overline{\mathrm{T}}}{\alpha}\right). \qquad (7c)$$

It can be seen that the dimensionless Eq. 6 and 7 are the same as the equations of the dimensionless TL model in our original manuscript (Eq. 7 and Eq. 8). Therefore, technically this does not affect the comparison between simulation and experiment in our work. The essential reason is that the charging behavior of the ionic liquid in the nanopore and the behavior described by the TL model are both in the form of diffusion, as discussed above. Hence, the diffusion model derived here can be used as a model to describes the (dis)charging of the ionic liquids in the nanopore and bridge molecular simulations and experiments in our work.

Brief summary:
Based on the reviewer's insightful suggestion, the above discussions and new derivation of the diffusion model have been incorporated into the revised manuscript (*Pages* **8-9**, *lines* **174-189**; **Methods** section, *Pages* **14-16**, *lines* **335-364**), with the sketch of the diffusion model added into Supplementary Information as **Supplementary Figure 21** (*Page* **22**).

Furthermore, we also agree with what the reviewer that: "I just feel that the claims are bit exaggerated when the authors say "dynamics are in excellent agreement", when they rely on another model with its own limitations.", thus we weaken the exaggerated statement by deleting "excellent".

Specifically, the modification in the main text is:
"The charging dynamics of nanoporous electrodes can be illuminated by the diffusion model from the mean-field theory[12, 38]. To bridge macroscale experiments and nanoscale modeling, we derive the dimensionless form of the diffusion model for the charging and discharging process…. Details of the dimensionless diffusion model can

be found in Methods. As shown in Fig. 4a, the dimensionless diffusion model provides … bridged by the dimensionless diffusion model." (*Pages* **8-9**, *lines* **174-189**)

The modification in the **Methods** section is:

"Diffusion model for galvanostatic charge-discharge

As mentioned above, the charging-discharging process of ionic liquids in a nanopore can be described by the charge diffusion equation (Eq. 1) derived from mean-field theory[12, 38]. For the galvanostatic charge-discharge, the initial and boundary conditions are set as:

$$\rho_e|_{t=0} = 0, \tag{5a}$$

$$\left.\frac{\partial \rho_e}{\partial z}\right|_{z=l} = 0, \tag{5b}$$

$$\left.\frac{\partial \phi}{\partial z}\right|_{z=0} = -\frac{I_0}{D_{eff}} S\left(\frac{t}{P}\right), \tag{5c}$$

where l is pore length, and S is a square-wave function whose period and amplitude are both one. Equation 5a indicates the pore is uncharged at $t = 0$. The boundary condition in Eq. 5b is the zero normal flux at $z = l$ (corresponds to the middle position of the open pore in our simulations), and the boundary in Eq. 5c indicates the applied square-wave current with a period P and an amplitude I_0, as shown in Fig. 1d.

As shown in Supplementary Fig. 21, the ions are distributed in the nanopore, and the electrode is equipotential. Taking the areal capacitance, C, along the nanopore as a constant, we divide the pore into numerous micro-capacitors in parallel with each having the capacitance of Cdz. Then we can get

$$\rho_e(z, t)dz = [\phi_1(z, t) - \phi_0(t)]Cdz, \tag{6}$$

where $\phi_1(z, t)$ is the potential in the electrolyte and $\phi_0(t)$ is the potential on the electrode. Using the notation for the potential drop from the electrolyte to electrode $\phi(z, t) = \phi_1(z, t) - \phi_0(t)$ and substituting Eq. 6 into Eq. 1 and Eq. 5, we, respectively, obtain the diffusion equation for potential

$$\frac{\partial \phi}{\partial t} = D_{eff} \frac{\partial \phi^2}{\partial^2 z}, \tag{7}$$

and initial and boundary conditions

$$\phi|_{t=0} = 0, \tag{8a}$$

$$\left.\frac{\partial \phi}{\partial z}\right|_{z=l} = 0, \tag{8b}$$

$$\left.\frac{\partial \phi}{\partial z}\right|_{z=0} = -\frac{I_0}{CD_{eff}} S\left(\frac{t}{P}\right). \tag{8c}$$

It is worth noting that Eq. 7 and Eq. 8 are in the same form as the transmission line model[67, 68], since the charging process predicted by the transmission model also exhibits diffusive behavior[12]. We define the dimensionless potential $\bar{\Phi} = \frac{CD_{eff}}{I_0 l}\phi$, dimensionless position $Z = z/l$, dimensionless time $\bar{T} = t/\tau$ ($\tau = l^2/D_{eff}$ is the charging time constant) (**Methods** section, *Pages* **14-16**, *lines* **335-364**)

The modification in the Supplementary Information is:

"

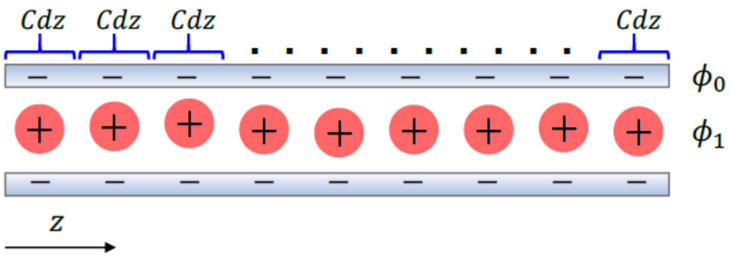

Supplementary Figure 21 | Sketch of the diffusion model for the charging of a nanopore filled with ionic liquids.

" (**Part 10**, *Pages* **21**)

2. One of the interesting features that authors can calculate in their systems is the effective properties. While the effective diffusivities have been reported, could authors also extract a capacitance value? I think it would be very instructive to see the "absolute" capacitance value from MD simulations. For instance, it is well known that experimentally observed capacitance values are on the order of O(0.1) F/m^2 …It would be instructive to know whether this is followed in the simulations reported here.

Response:
We thank the reviewer for these constructive comments. Herein, following the common practice in experimental studies, we extracted areal capacitance from GCD curves by[23]

$$C = 2\frac{I\Delta t}{A\Delta V}, \quad (8)$$

where C is the areal capacitance of single electrode, I is the constant current, Δt is the discharging time, A is the surface area of the electrode, and ΔV is the voltage change during the discharge (the voltage change removes IR drop for experiments). As shown in **Figure R3.2**, the capacitance obtained by MD simulations of GCD-CPM is 1.0~1.8 $\mu F/cm^2$ with dimensionless current period α from 0.42 to 1.75, and the experimental capacitance measured in the experiment is 1.3~2.8 $\mu F/cm^2$ with the same dimensionless current periods (correspondingly, the current density is 1 to 0.1 A/g). Therefore, capacitances from simulations and experiments are comparable, and both increase with the increase of the current period.

It is worth noting that although the capacitance is not as large as ~10 $\mu F/cm^2$ as mentioned by the reviewer, it is compatible with previous experimental measurements using the same type of carbon electrodes (i.e., activated carbon cloths) and electrolyte [EMIM][BF_4]. For instance, the areal capacitance measured by GCD tests in the previous experiment[24], which taking activated carbon cloth as the electrode and [EMIM][BF_4] as the electrolyte, is 1.57~1.81 $\mu F/cm^2$ with the current density from 1 to 0.1 A/g (results shown in Figure 2 of this reference is 28.3~32.6 F/g, and the electrode specific surface is 1800 m^2/g). Similarly, in the another experimental work of supercapacitors with activated carbon cloth with the same electrolyte[25], the areal capacitance measured by CV tests was 3.49 $\mu F/cm2$ at a scan rate of 5 mV/s (Table 1 of this reference shows that the capacitance is 0.55 F, and the mass of and specific surface area of the electrode is 6.3 mg and 2500 $m2/g$).

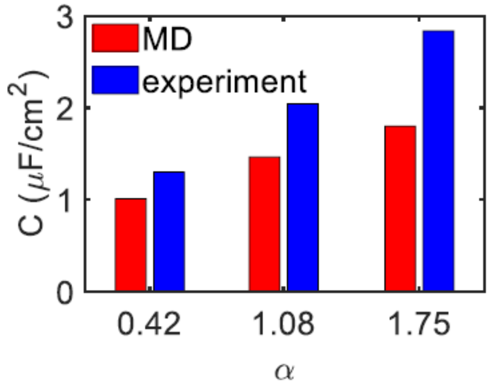

Figure R3.2 | Areal capacitances extracted from GCD curves obtained by molecular

simulations of GCD-CPM and experiments.

Brief summary:
Based on the reviewer's suggestion, the above discussion and new data have been incorporated into the revised manuscript (*Page* **9**, *lines* **192-195**; **Supplementary Part 7** with **Supplementary Figure 15** of Supplementary Information, *Pages* **14-15**).

Specifically, the modification in the main text is:
"Furthermore, the capacitances extracted from GCD curves obtained from molecular simulations with GCD-CPM are compatible with those from our experimental measurements (Supplementary Fig. 15), both consistent with previous experiments of the similar porous carbon electrode with the same electrolyte[41,42]." (*Page* **9**, *lines* **192-195**)
New references are added:
"41. Lewandowski A., Olejniczak A., Galinski M., Stepniak I. Performance of carbon–carbon supercapacitors based on organic, aqueous and ionic liquid electrolytes. *Journal of Power Sources* 2010, 195(17)**:** 5814-5819.
42. Yamazaki S., Ito T., Yamagata M., Ishikawa M. Non-aqueous electrochemical capacitor utilizing electrolytic redox reactions of bromide species in ionic liquid. *Electrochimica Acta* 2012, 86**:** 294-297."

The modification in the Supplementary Information is:
"Areal capacitances extracted from GCD curves are obtained by[6]

$$C = 2\frac{I\Delta t}{A\Delta V}, \quad \text{(S2)}$$

where C is the areal capacitance of single electrode, I is the constant current, Δt is the discharging time, A is the surface area of the electrode, and ΔV is the voltage change during the discharge (the voltage change removes IR drop for experiments). The areal capacitances obtained by simulations and experiments are shown in Supplementary Fig. 15…

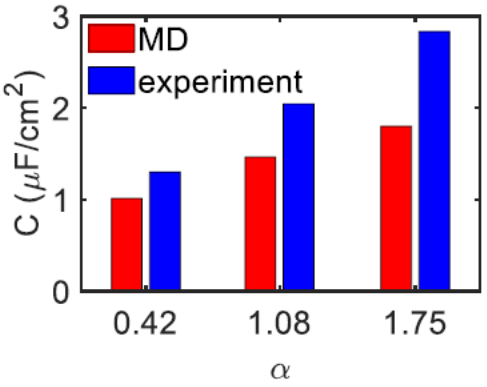

Supplementary Figure 15 | Areal capacitances extracted from GCD curves obtained by simulations and experiments." **(Part 7**, *Pages* **14-15**).

3. Building up on 2., could authors also comment on the effect of capacitance on pore size from their simulations? This is a hotly debated topic in the supercaps community where claims have suggested specific capacitance can decrease with increase in pore size and can also stay the same. Therefore, it would be good to know if the authors can show such dependence from their simulations.

Response:
We thank the reviewer for these insightful comments. The dependence of capacitance on pore size has indeed been a hotly debated topic. While some experiments showed that, in certain pore size ranges, capacitance increases as pore size decreases (this phenomenon is often termed "anomalous enhancement")[19, 26, 27], some experiment does not support such anomalous enhancement[28-30]. The dependence of capacitance on pore size has been studied extensively in the theoretical[31, 32] and simulation community[33-37], including us[33, 36, 37].

[*REDACTED*]

Figure R3.3 | Effect of pore size on capacitance[34-37]. a, Relation between capacitance and pore size of ionic liquid in nanopores obtained by classic density functional theory, the figure is copied from Ref. 34. **b**, Dependence of capacitance on pore size and roughness of electrodes obtained by molecular simulations with constant potential; the figure is copied from Ref. 35.**c,** The capacitance of the ionic liquid in the nanopore as a function of the pore size obtained by molecular dynamics, the figure is copied from Ref. 36. **d,** The effect of pore size on the capacitance and charging speed of ionic liquid in nanopores obtained by molecular simulations with constant potential; the figure is copied from Ref. 37.

Among these studies, Jiang *et al.*[34] found that the capacitance of ionic liquid in nanopores oscillated with a decaying envelope as the pore size increases and attributed the oscillating behavior to the interference of the overlapping electric double layers, using classic density functional theory (**Figure R3.3 a**). Vatamanu *et al.*[35] used constant potential MD simulations to study the dependence of capacitance on pore size and roughness of electrodes in several systems with various carbon-based nanoporous electrodes (*e.g.*, slit pores, cylindrical pores, and slit pores with rough walls) and ionic liquid electrolytes (*e.g.*, [pyr14][FSI], [C_2mim][TFSI], [C_4mim][TFSI],[C_8mim][TFSI], and [C_4mim][PF_6]). They show that the capacitance increases as the pore size decreases, and the roughness of the electrode also enhances the capacitance(**Figure R3.3b**). We used MD simulations to explore the capacitance of slit-shaped nanopores with ionic liquid and found that capacitance exhibits a U-shaped scaling curve behavior with pore widths from 0.75 to 1.26 nm, in which the left branch of the capacitance scaling curve corresponds to the capacitance increase[36] (**Figure R3.3c**).

Recently, we performed MD simulations with the constant potential method to investigate the capacitance and charging dynamic of the ionic liquid [EMIM][BF_4] in the nanopores with different pore sizes. We not only showed the relation between the capacitance and pore size but also found that the charging of nanopore could be speeded up by the in-pore ion structure transition (**Figure R3.3d**). In fact, our GCD-CPM technique in this work is developed based on the previous work (i.e., the potentiostatic mode)[37].

While the enhancement of capacitance over a certain range of pore size is generally supported by theoretical and computational studies, we also note that the level of enhancement differs from study to study and can be quite modest. This, along with the fact that any size dependence of capacitance tends to be smeared out by any nonuniformity in electrodes' pore size, may explain why some experiments show no obvious capacitance enhancement[38].

Herein, we briefly discussed the relation between pore size and capacitance, as the reviewer suggested, which is incorporated into the revised manuscript (**Discussion** section of the main text, *Page* **12**, *lines* **260-263**).

The modification is:

"In particular, the dependence of capacitance on pore size has been intensively investigated by molecular simulations in the potentiostatic mode[3, 49-51]. Such dependence of charging dynamics, especially in the galvanostatic mode, is also significant, and can be investigated by GCD-CPM." (**Discussion** section of the main text, *Page* **12**, *lines* **260-263**).

New references are added:

"49. Wu P., Huang J., Meunier V., Sumpter B. G., Qiao R. Complex capacitance scaling in ionic liquids-filled nanopores. ACS Nano 2011, 5(11): 9044-9051.

50. Vatamanu J., Vatamanu M., Bedrov D. Non-Faradaic energy storage by room temperature ionic liquids in nanoporous electrodes. *ACS Nano* 2015, 9(6): 5999-6017.
51. Mo T., Bi S., Zhang Y., Presser V., Wang X., Gogotsi Y., Feng G. Ion structure transition enhances charging dynamics in subnanometer pores. *ACS Nano* 2020, 14(2): 2395-2403."

References

1. Evans D. J., Holian B. L. The Nose–Hoover thermostat. *The Journal of Chemical Physics* 1985, 83(8): 4069-4074.
2. Berendsen H. J. C. Transport properties computed by linear response through weak coupling to a bath. In: Meyer Madeleine, Pontikis Vassilis (eds). *Computer simulation in materials science: Interatomic potentials, simulation techniques and applications*. Springer Netherlands: Dordrecht, 1991pp 139-155.
3. Bussi G., Donadio D., Parrinello M. Canonical sampling through velocity rescaling. *The Journal of Chemical Physics* 2007, 126(1): 014101.
4. Esposito M., Monnai T. Nonequilibrium thermodynamics and Nose−Hoover dynamics. *The Journal of Physical Chemistry B* 2011, 115(18): 5144-5147.
5. Pak A. J., Hwang G. S. Charging rate dependence of ion migration and stagnation in ionicliquid-filled carbon nanopores. *The Journal of Physical Chemistry C* 2016, 120(43): 24560-24567.
6. Merlet C., Pean C., Rotenberg B., Madden P. A., Simon P., Salanne M. Simulating supercapacitors: Can we model electrodes as constant charge surfaces? *Journal of Physical Chemistry Letters* 2013, 4(2): 264-268.
7. Darden T., York D., Pedersen L. Particle mesh Ewald: An n·log(n) method for Ewald sums in large systems. *The Journal of Chemical Physics* 1993, 98(12): 10089-10092.
8. Yeh I.-C., Berkowitz M. L. Ewald summation for systems with slab geometry. *The Journal of Chemical Physics* 1999, 111(7): 3155-3162.
9. Scalfi L., Salanne M., Rotenberg B. Molecular simulation of electrode-solution interfaces. *Annual Review of Physical Chemistry* 2021, 72(1): 189-212.
10. Fedorov M. V., Kornyshev A. A. Ionic liquid near a charged wall: Structure and capacitance of electrical double layer. *The Journal of Physical Chemistry B* 2008, 112(38): 11868-11872.
11. Zhang Y., Cummings P. T. Effects of solvent concentration on the performance of ionicliquid/carbon supercapacitors. *ACS Applied Materials & Interfaces* 2019, 11(45): 42680-42689.
12. Zhan C., Zhang Y., Cummings P. T., Jiang D. Computational insight into the capacitive performance of graphene edge planes. *Carbon* 2017, 116: 278-285.
13. De Levie R. On porous electrodes in electrolyte solutions: I. Capacitance effects. *Electrochimica Acta* 1963, 8(10): 751-780.
14. Gupta A., Zuk P. J., Stone H. A. Charging dynamics of overlapping double layers in a cylindrical nanopore. *Physical Review Letters* 2020, 125(7): 076001.
15. Pean C., Merlet C., Rotenberg B., Madden P. A., Taberna P. L., Daffos B., Salanne M., Simon P. On the dynamics of charging in nanoporous carbon-based supercapacitors. *ACS Nano* 2014, 8(2): 1576-1583.
16. Bi S., Banda H., Chen M., Niu L., Chen M., Wu T., Wang J., Wang R., Feng J., Chen T.,

Dincă M., Kornyshev A. A., Feng G. Molecular understanding of charge storage and charging dynamics in supercapacitors with MOF electrodes and ionic liquid electrolytes. *Nature Materials* 2020, 19(5): 552-558.
17. Gan Z., Wang Y., Wang M., Gao E., Huo F., Ding W., He H., Zhang S. Ionophobic nanopore enhancing capacitance and charging dynamics in supercapacitor with ionic liquids. *Journal of Materials Chemistry A* 2021.
18. Gourdin G., Meehan A., Jiang T., Smith P., Qu D. Investigation of the impact of stacking pressure on a double-layer supercapacitor. *Journal of Power Sources* 2011, 196(1): 523-529.
19. Largeot C., Portet C., Chmiola J., Taberna P.-L., Gogotsi Y., Simon P. Relation between the ion size and pore size for an electric double-layer capacitor. *Journal of the American Chemical Society* 2008, 130(9): 2730-2731.
20. Qu D., Shi H. Studies of activated carbons used in double-layer capacitors. *Journal of Power Sources* 1998, 74(1): 99-107.
21. Kondrat S., Wu P., Qiao R., Kornyshev A. A. Accelerating charging dynamics in subnanometre pores. *Nature Materials* 2014, 13(4): 387-393.
22. Kondrat S., Kornyshev A. Charging dynamics and optimization of nanoporous supercapacitors. *The Journal of Physical Chemistry C* 2013, 117(24): 12399-12406.
23. Zhang S., Pan N. Supercapacitors performance evaluation. *Advanced Energy Materials* 2015, 5(6): 1401401.
24. Yamazaki S., Ito T., Yamagata M., Ishikawa M. Non-aqueous electrochemical capacitor utilizing electrolytic redox reactions of bromide species in ionic liquid. *Electrochimica Acta* 2012, 86: 294-297.
25. Lewandowski A., Olejniczak A., Galinski M., Stepniak I. Performance of carbon–carbon supercapacitors based on organic, aqueous and ionic liquid electrolytes. *Journal of Power Sources* 2010, 195(17): 5814-5819.
26. Chmiola J., Yushin G., Gogotsi Y., Portet C., Simon P., Taberna P. L. Anomalous increase in carbon capacitance at pore sizes less than 1 nanometer. *Science* 2006, 313(5794): 1760-1763.
27. Raymundo-Piñero E., Kierzek K., Machnikowski J., Béguin F. Relationship between the nanoporous texture of activated carbons and their capacitance properties in different electrolytes. *Carbon* 2006, 44(12): 2498-2507.
28. Centeno T. A., Sereda O., Stoeckli F. Capacitance in carbon pores of 0.7 to 15 nm: A regular pattern. *Physical Chemistry Chemical Physics* 2011, 13(27): 12403-12406.
29. García-Gómez A., Moreno-Fernández G., Lobato B., Centeno T. A. Constant capacitance in nanopores of carbon monoliths. *Physical Chemistry Chemical Physics* 2015, 17(24): 15687-15690.
30. Stoeckli F., Centeno T. A. Pore size distribution and capacitance in microporous carbons. *Physical Chemistry Chemical Physics* 2012, 14(33): 11589-11591.
31. Kondrat S., Georgi N., Fedorov M. V., Kornyshev A. A. A superionic state in nano-porous double-layer capacitors: Insights from monte carlo simulations. *Physical Chemistry Chemical Physics* 2011, 13(23): 11359-11366.
32. Lian Z., Chao H., Wang Z.-G. Effects of confinement and ion adsorption in ionic liquid supercapacitors with nanoporous electrodes. *ACS Nano* 2021, 15(7): 11724-11733.

33. Feng G., Cummings P. T. Supercapacitor capacitance exhibits oscillatory behavior as a function of nanopore size. *The Journal of Physical Chemistry Letters* 2011, 2(22): 2859-2864.
34. Jiang D., Jin Z., Wu J. Oscillation of capacitance inside nanopores. *Nano Letters* 2011, 11(12): 5373-5377.
35. Vatamanu J., Vatamanu M., Bedrov D. Non-Faradaic energy storage by room temperature ionic liquids in nanoporous electrodes. *ACS Nano* 2015, 9(6): 5999-6017.
36. Wu P., Huang J., Meunier V., Sumpter B. G., Qiao R. Complex capacitance scaling in ionic liquids-filled nanopores. *ACS Nano* 2011, 5(11): 9044-9051.
37. Mo T., Bi S., Zhang Y., Presser V., Wang X., Gogotsi Y., Feng G. Ion structure transition enhances charging dynamics in subnanometer pores. *ACS Nano* 2020, 14(2): 2395-2403.
38. Jäckel N., Rodner M., Schreiber A., Jeongwook J., Zeiger M., Aslan M., Weingarth D., Presser V. Anomalous or regular capacitance? The influence of pore size dispersity on double-layer formation. *Journal of Power Sources* 2016, 326: 660-671.

Decision Letter, first revision:

Dear Dr. Feng,

Thank you for your patience as we've prepared the guidelines for final submission of your Nature Computational Science manuscript, "Modeling galvanostatic charge-discharge of nanoporous supercapacitors" (NATCOMPUTSCI-21-0397A). Please carefully follow the step-by-step instructions provided in the attached file, and add a response in each row of the table to indicate the changes that you have made. Please also check and comment on any additional marked-up edits we have proposed within the text. Ensuring that each point is addressed will help to ensure that your revised manuscript can be swiftly handed over to our production team.

We would like to start working on your revised paper, with all of the requested files and forms, as soon as possible (preferably within 5-7 days). Please get in contact with us if you anticipate delays.

When you upload your final materials, please include a point-by-point response to any remaining reviewer comments.

If you have not done so already, please alert us to any related manuscripts from your group that are under consideration or in press at other journals, or are being written up for submission to other journals (see: https://www.nature.com/nature-research/editorial-policies/plagiarism#policy-on-duplicate-publication for details).

In recognition of the time and expertise our reviewers provide to Nature Computational Science's editorial process, we would like to formally acknowledge their contribution to the external peer review of your manuscript entitled "Modeling galvanostatic charge-discharge of nanoporous supercapacitors".

For those reviewers who give their assent, we will be publishing their names alongside the published article.

Cover suggestions

As you prepare your final files we encourage you to consider whether you have any images or illustrations that may be appropriate for use on the cover of Nature Computational Science.

Covers should be both aesthetically appealing and scientifically relevant, and should be supplied at the best quality available. Due to the prominence of these images, we do not generally select images featuring faces, children, text, graphs, schematic drawings, or collages on our covers.

We accept TIFF, JPEG, PNG or PSD file formats (a layered PSD file would be ideal), and the image should be at least 300ppi resolution (preferably 600-1200 ppi), in CMYK colour mode.

If your image is selected, we may also use it on the journal website as a banner image, and may need to make artistic alterations to fit our journal style.

Please submit your suggestions, clearly labeled, along with your final files. We'll be in touch if more information is needed.

Nature Computational Science has now transitioned to a unified Rights Collection system which will allow our Author Services team to quickly and easily collect the rights and permissions required to publish your work. Approximately 10 days after your paper is formally accepted, you will receive an email in providing you with a link to complete the grant of rights. If your paper is eligible for Open Access, our Author Services team will also be in touch regarding any additional information that may be required to arrange payment for your article.

Please note that you will not receive your proofs until the publishing agreement has been received through our system.

Please note that <i>Nature Computational Science</i> is a Transformative Journal (TJ). Authors may publish their research with us through the traditional subscription access route or make their paper immediately open access through payment of an article-processing charge (APC). Authors will not be required to make a final decision about access to their article until it has been accepted. Find out more about Transformative Journals

Authors may need to take specific actions to achieve <a

href="https://www.springernature.com/gp/open-research/funding/policy-compliance-faqs"> compliance with funder and institutional open access mandates. For submissions from January 2021, if your research is supported by a funder that requires immediate open access (e.g. according to Plan S principles) then you should select the gold OA route, and we will direct you to the compliant route where possible. For authors selecting the subscription publication route our standard licensing terms will need to be accepted, including our self-archiving policies. Those standard licensing terms will supersede any other terms that the author or any third party may assert apply to any version of the manuscript.

For information regarding our different publishing models please see our Transformative Journals page. If you have any questions about costs, Open Access requirements, or our legal forms, please contact ASJournals@springernature.com.

Please use the following link for uploading these materials:

[***REDACTED***]

** Please ensure you delete the link to your author homepage in this e-mail if you wish to forward it to your co-authors. **

If you have any further questions, please feel free to contact me.

Best regards,

Editorial Assistant
Nature Computational Science

On behalf of
Jie Pan, Ph.D.
Associate Editor
Nature Computational Science

Reviewer #1:
Remarks to the Author:

The authors have revised their manuscript in response to the reviewer concerns. Their response has been thorough and the manuscript is now in suitable form for publication.

Reviewer #2:
Remarks to the Author:
I believe in the revised version the authors adequately addressed all comments raised by the reviewers. I believe the manuscript is ready for publication in current form.

Reviewer #3:
Remarks to the Author:
I congratulate the authors for comprehensively addressing all my comments. I enjoyed reading this paper and believe it will be well-received by the community.

Author Rebuttal, first revision:

We've made point-to-point response to each comment. Specifically, reviewers' comments are copied in blue, and each comment is followed by our response in black.

REVIEWERS' COMMENTS:

Reviewer #1 (Remarks to the Author):
The authors have revised their manuscript in response to the reviewer concerns. Their response has been thorough and the manuscript is now in suitable form for publication.

Response: We appreciate the reviewer for her/his time and work on re-reviewing our work and the recommendation for publication.

Reviewer #2 (Remarks to the Author):
I believe in the revised version the authors adequately addressed all comments raised by the reviewers. I believe the manuscript is ready for publication in current form.

Response: We thank this reviewer for her/his positive comment and recommendation for publication.

Reviewer #3 (Remarks to the Author):
I congratulate the authors for comprehensively addressing all my comments. I enjoyed

reading this paper and believe it will be well-received by the community.

Response: We appreciate the reviewer very much for her/his positive comment and enjoying our work.

Final Decision Letter:

Dear Professor Feng,

We are pleased to inform you that your Article "Modeling galvanostatic charge-discharge of nanoporous supercapacitors" has now been accepted for publication in Nature Computational Science.

In approximately 10 business days you will receive an email with a link to choose the appropriate publishing options for your paper and our Author Services team will be in touch regarding any additional information that may be required.

Please note that *Nature Computational Science* is a Transformative Journal (TJ). Authors may publish their research with us through the traditional subscription access route or make their paper immediately open access through payment of an article-processing charge (APC). Authors will not be required to make a final decision about access to their article until it has been accepted. Find out more about Transformative Journals

Authors may need to take specific actions to achieve compliance with funder and institutional open access mandates. For submissions from January 2021, if your research is supported by a funder that requires immediate open access (e.g. according to Plan S principles) then you should select the gold OA route, and we will direct you to the compliant route where possible. For authors selecting the subscription publication route our standard licensing terms will need to be accepted, including our self-archiving policies. Those standard licensing terms will supersede any other terms that the author or any third party may assert apply to any version of the manuscript.

You will not receive your proofs until the publishing agreement has been received through our system.

If you have any questions about our publishing options, costs, Open Access requirements, or our legal forms, please contact ASJournals@springernature.com

Acceptance of your manuscript is conditional on all authors' agreement with our publication policies (see https://www.nature.com/natcomputsci/for-authors). In particular your manuscript must not be

published elsewhere and there must be no announcement of the work to any media outlet until the publication date (the day on which it is uploaded onto our web site).

Before your manuscript is typeset, we will edit the text to ensure it is intelligible to our wide readership and conforms to house style. We look particularly carefully at the titles of all papers to ensure that they are relatively brief and understandable.

Once your manuscript is typeset and you have completed the appropriate grant of rights, you will receive a link to your electronic proof via email with a request to make any corrections within 48 hours. If, when you receive your proof, you cannot meet this deadline, please inform us at rjsproduction@springernature.com immediately.

If you have queries at any point during the production process then please contact the production team at rjsproduction@springernature.com. Once your paper has been scheduled for online publication, the Nature press office will be in touch to confirm the details.

Content is published online weekly on Mondays and Thursdays, and the embargo is set at 16:00 London time (GMT)/11:00 am US Eastern time (EST) on the day of publication. If you need to know the exact publication date or when the news embargo will be lifted, please contact our press office after you have submitted your proof corrections. Now is the time to inform your Public Relations or Press Office about your paper, as they might be interested in promoting its publication. This will allow them time to prepare an accurate and satisfactory press release. Include your manuscript tracking number NATCOMPUTSCI-21-0397B and the name of the journal, which they will need when they contact our office.

About one week before your paper is published online, we shall be distributing a press release to news organizations worldwide, which may include details of your work. We are happy for your institution or funding agency to prepare its own press release, but it must mention the embargo date and Nature Methods. Our Press Office will contact you closer to the time of publication, but if you or your Press Office have any inquiries in the meantime, please contact press@nature.com.

An online order form for reprints of your paper is available at https://www.nature.com/reprints/author-reprints.html. All co-authors, authors' institutions and authors' funding agencies can order reprints using the form appropriate to their geographical region.

We welcome the submission of potential cover material (including a short caption of around 40 words) related to your manuscript; suggestions should be sent to Nature Computational Science as electronic files (the image should be 300 dpi at 210 x 297 mm in either TIFF or JPEG format). We also welcome suggestions for the Hero Image, which appears at the top of our home page; these should be 72 dpi at

1400 x 400 pixels in JPEG format. Please note that such pictures should be selected more for their aesthetic appeal than for their scientific content, and that colour images work better than black and white or grayscale images. Please do not try to design a cover with the Nature Computational Science logo etc., and please do not submit composites of images related to your work. I am sure you will understand that we cannot make any promise as to whether any of your suggestions might be selected for the cover of the journal.

You can now use a single sign-on for all your accounts, view the status of all your manuscript submissions and reviews, access usage statistics for your published articles and download a record of your refereeing activity for the Nature journals.

To assist our authors in disseminating their research to the broader community, our SharedIt initiative provides you with a unique shareable link that will allow anyone (with or without a subscription) to read the published article. Recipients of the link with a subscription will also be able to download and print the PDF.

As soon as your article is published, you will receive an automated email with your shareable link.

We look forward to publishing your paper.

Best regards,

Jie Pan, Ph.D.
Associate Editor
Nature Computational Science
